# Sexual Antagonism, Temporally Fluctuating Selection, and Variable Dominance Affect a Regulatory Polymorphism in *Drosophila melanogaster*

**DOI:** 10.1093/molbev/msab215

**Published:** 2021-07-21

**Authors:** Amanda Glaser-Schmitt, Meike J Wittmann, Timothy J S Ramnarine, John Parsch

**Affiliations:** 1 Division of Evolutionary Biology, Faculty of Biology, Ludwig-Maximilians-University Munich, Planegg-Martinsried, Germany; 2 Faculty of Biology, Department of Theoretical Biology, Bielefeld University, Bielefeld, Germany

**Keywords:** regulatory evolution, balancing selection, sexual conflict, seasonal variation, X chromosome, phenotypic variation

## Abstract

Understanding how genetic variation is maintained within species is a major goal of evolutionary genetics that can shed light on the preservation of biodiversity. Here, we examined the maintenance of a regulatory single-nucleotide polymorphism (SNP) of the X-linked *Drosophila melanogaster* gene *fezzik*. The derived variant at this site is at intermediate frequency in many worldwide populations but absent in populations from the ancestral species range in sub-Saharan Africa. We collected and genotyped wild-caught individuals from a single European population biannually over a period of 5 years, which revealed an overall difference in allele frequency between the sexes and a consistent change in allele frequency across seasons in females but not in males. Modeling based on the observed allele and genotype frequencies suggested that both sexually antagonistic and temporally fluctuating selection may help maintain variation at this site. The derived variant is predicted to be female-beneficial and mostly recessive; however, there was uncertainty surrounding our dominance estimates and long-term modeling projections suggest that it is more likely to be dominant. By examining gene expression phenotypes, we found that phenotypic dominance was variable and dependent upon developmental stage and genetic background, suggesting that dominance may be variable at this locus. We further determined that *fezzik* expression and genotype are associated with starvation resistance in a sex-dependent manner, suggesting a potential phenotypic target of selection. By characterizing the mechanisms of selection acting on this SNP, our results improve our understanding of how selection maintains genetic and phenotypic variation in natural populations.

## Introduction

Genetic variation is an essential component of evolution and is shaped by neutral and selective forces that drive changes in allele frequency, leading to the maintenance, loss, or fixation of individual genetic variants over time. How variation is maintained over extended periods of time remains a topic of intense interest and ongoing study in evolutionary biology (Charlesworth 2006; Leffler et al. 2012; Key et al. 2014; Connallon and Clark 2014a; Koenig et al. 2019; Chapman et al. 2019). Elucidating the forces that preserve genetic polymorphism in a population or species can help us to understand the mechanisms underlying the maintenance of biodiversity and potentially help in the management of endangered species and ecosystems.

Balancing selection can maintain polymorphism in a population through several, nonmutually exclusive mechanisms. Perhaps the best-known form of balancing selection is overdominance, which is also known as heterozygote advantage because it refers to situations where the heterozygote has a higher fitness than either homozygote. It has been predicted that overdominant selection may be common when there is frequent adaptation, as large-effect adaptive variants may overshoot the fitness optimum in their homozygous state but meet the optimum in their heterozygous state (Sellis et al. 2011). Genetic variation can also be maintained if the selective effect of alternate alleles varies over time or across space, though in both cases the presence of heterogeneous selection is not a sufficient criterion for maintenance of polymorphism and additional conditions need to be met. For instance, theoretical and empirical studies have found evidence that temporally varying selection can maintain polymorphism within populations via regular, seasonal allele frequency fluctuations of selected and linked variants (Bergland et al. 2014; Wittmann et al. 2017; but see Buffalo and Coop 2020), whereas spatially varying selection across latitude, longitude, or altitude can lead to stable clines of allele frequencies (Fabian et al. 2015; Kapun et al. 2016; Durmaz et al. 2018). Another mechanism that can maintain genetic diversity is genomic conflict. Sexual antagonism, for example, occurs when the fitness optimum of a trait differs between the sexes. Sexual antagonism is thought to be pervasive in natural populations (Innocenti and Morrow 2010; Cheng and Kirkpatrick 2016) and to play an important role in the maintenance of polymorphism (Connallon and Clark 2014a, 2014b; Mank 2017; Ruzicka et al. 2019) as well as drive divergence between populations and species (Payseur et al. 2018; Lund-Hansen et al. 2021).

For selection to act upon a genetic variant and alter its frequency, the variant must affect an organismal phenotype that, in turn, must affect fitness. Gene expression variation underlies much of the phenotypic variation that is observed within and among populations and species (King and Wilson 1975; Wray et al. 2003). Driving this expression variation are the causal regulatory variants that modulate a gene’s expression level and are exposed to selection when they affect an organism’s fitness (reviewed by Hill et al. [2021]). Indeed, regulatory variants are thought to be particularly important during adaption because changes in the timing and breadth of gene expression are less likely to have deleterious pleiotropic effects than protein-coding changes (Carroll 2000, 2008). Although some advantageous regulatory variants may sweep to fixation or be lost during adaptation, others may be maintained alongside alternate alleles in a population by balancing selection. Thus, determining the trait under selection and the effects of a selected regulatory variant on organismal fitness is extremely challenging.

The *Drosophila melanogaster* gene *fezzik* (*fiz*) is located on the X chromosome and predicted to have oxidoreductase activity (Gramates et al. 2017) as well as play a role in ecdysteroid metabolism (Iida et al. 2007). It is involved in larval growth and body size determination, although its expression has also been shown to affect insecticide and cold tolerance (Glaser-Schmitt and Parsch 2018). The expression of *fiz* is typically 2–5 times higher in populations outside of sub-Saharan Africa (here referred to as cosmopolitan) than in sub-Saharan African populations (Glaser-Schmitt and Parsch 2018). This expression divergence has been mapped to a 1.2-kb upstream regulatory region referred to as the *fiz* enhancer (Saminadin-Peter et al. 2012; Glaser-Schmitt and Parsch 2018). Previous studies found evidence that the *fiz* enhancer was a target of positive selection in cosmopolitan populations (Saminadin-Peter et al. 2012; Glaser-Schmitt et al. 2013), suggesting a beneficial effect of increased *fiz* expression as *D. melanogaster* expanded out of its ancestral range in sub-Saharan Africa (Glaser-Schmitt et al. 2013). Within the *fiz* enhancer a single-nucleotide polymorphism (SNP) located 67 bases upstream of the start codon (referred to here as position 67) was found to have a major effect on *fiz* expression (Glaser-Schmitt and Parsch 2018). At this position, two nucleotides segregate in natural populations: a derived cosmopolitan “G” variant (henceforth G), which is associated with increased *fiz* expression, and an ancestral sub-Saharan African “C” variant (henceforth C). Other cosmopolitan regulatory variants in the *fiz* enhancer appear to have been fixed by positive selection in a selective sweep that occurred before position 67 became polymorphic, as position 67 is variable within an otherwise fixed haplotype spanning the *fiz* enhancer (Glaser-Schmitt and Parsch 2018).

In this study, we examined allele and genotype frequencies over the course of 5 years in a derived, European *D. melanogaster* population in order to better understand the mechanisms maintaining the polymorphism at position 67. We find empirical evidence for the influence of both sex and season on allele frequency. Using a modeling approach, we determined that sexually antagonistic, temporally varying selection, or likely both are acting on this SNP and could help maintain polymorphism at this site. We further examined gene expression and body-size phenotypes associated with variants at position 67 in order to assess dominance, which plays an important role in the dynamics of selection at a locus. We detected significant variation in dominance within a single trait (gene expression) dependent upon the developmental stage and genetic background, although phenotypic dominance estimates were generally in line with parameter estimates from our model. Indeed, these results suggest that variable dominance plays an important role in shaping allele frequency dynamics and this may help maintain polymorphism at position 67. Furthermore, we identified a novel association between starvation resistance and *fiz* expression. This association was sex-dependent, with increased female starvation resistance associated with the high-expression, derived G variant, and increased male starvation resistance associated with reduced *fiz* expression, suggesting that genetic variants underlying starvation resistance may be sexually antagonistic.

## Results

### Allele and Genotype Frequencies in Europe

The SNP at position 67 was previously identified experimentally as having a large effect on *fiz* expression and was found to segregate among isofemale lines derived from cosmopolitan populations (Glaser-Schmitt and Parsch 2018). To better characterize the allele frequencies at this site, we examined pooled whole genome sequencing (pool-seq) data of European populations collected by the European *Drosophila* Population Genetics Consortium (DrosEU) (Kapun et al. 2020; Kapun et al. 2021). Data from wild-caught flies collected in 47 European populations sampled at least once between 2014 and 2016 revealed that the derived G at position 67 was present at intermediate frequency in all surveyed populations (median = 36%; range = 12–66%; supplementary table S1, Supplementary Material online). The pool-seq data are limited, however, in that each pool consisted of only 40 males. To better examine how allele and genotype frequencies at position 67 change over time, we genotyped wild-caught male and female *D. melanogaster* from a derived population in Munich, Germany collected in June and September of each year from 2016 to 2020. For each collection, we genotyped 22–90 males and 84–132 females for a total of 515 males and 1,028 females over the course of 5 years (supplementary table S2, Supplementary Material online). For almost all collections, we obtained more females than males. The sex ratio of offspring from wild-caught females did not differ significantly from 50:50 (supplementary table S3, Supplementary Material online; Cochran–Mantel–Haenszel [CMH] test, *P *=* *0.764); thus, this imbalance is unlikely to be due to an unequal sex ratio immediately following reproduction. Instead, it may be a result of females’ increased attraction to food sources as potential oviposition sites resulting in more frequent trap visits in comparison to males. Alternatively, an increased rate of mortality in males before the time of sampling could also lead to their underrepresentation in our collections.

**Fig. 1.**
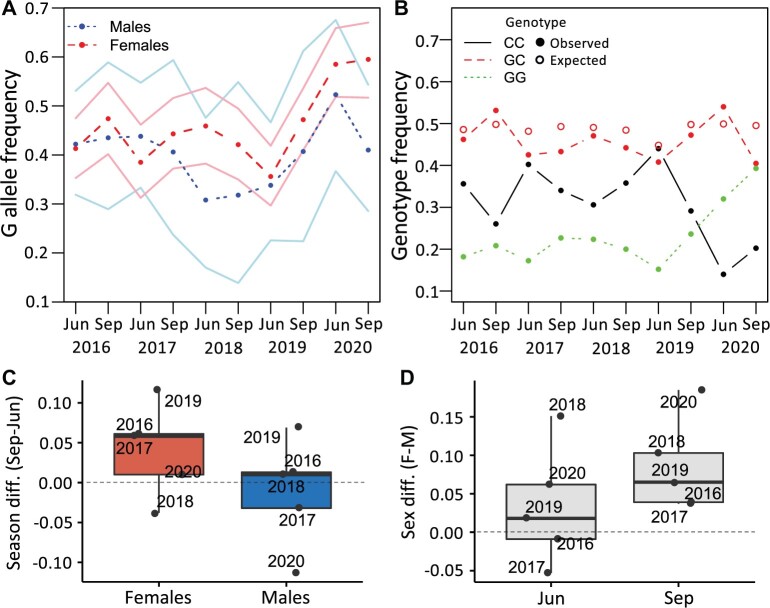
Variation of SNP 67 in Munich across seasons and years. (*A*) Male (blue) and female (red) frequency of the derived G allele for each collection. The 95% binomial confidence intervals are shown as light, solid lines. (*B*) Female genotype frequency for each collection. Red, open circles indicate expected GC frequency based on the allele frequency for all collected flies at each sampling point. (*C*) Difference (diff.) in G allele frequency between September (Sep) and June (Jun) in males and females. (*D*) Difference in G allele frequency between males (M) and females (F) for all collections.

The frequency of the G variant was higher in females than in males for eight of the ten collections, with the largest difference being 19% (in September 2020) and the average difference being 6% (fig. 1*A* and *D*; table 1). The difference between the sexes was significant by a bootstrapping test and marginally nonsignificant by the CMH test (table 1). In females, the frequency of the G variant generally showed larger seasonal fluctuations than in males (fig. 1*A* and *C*), with the variance in G frequency being 0.006 in females and 0.004 in males. The increased variance in females is unlikely to be the result of sampling error (bootstrapping test, *P *=* *0.046), because the sample sizes were consistently larger for females. In females, the G variant tended to increase in frequency between June and September and decrease between September and June (fig. 1*A* and *C*). Indeed, in females the frequency of the G allele was higher in September than in June for 4 of the 5 years (fig. 1*A* and *C*), with an average seasonal difference of 4%, which was significant by the CMH test and marginally nonsignificant by the bootstrapping test (table 1). There was no consistent seasonal pattern in males, where the frequency of the G allele was, on average, 1% higher in June than in September (fig. 1*A* and *C*) and did not differ significantly between seasons by either test (table 1). Thus, we detected a significant effect of sex on allele frequency across all seasons, but a significant effect of season only for females (fig. 1*A* and *C*; table 1). Note, however, that the larger sample sizes provided greater statistical power to detect seasonal effects in females. Given our male samples sizes, the power to detect a consistent seasonal difference in allele frequency of 4% at a *P*-value of 0.05 is only about 5%. The female genotype frequencies did not differ significantly from the expectations of Hardy–Weinberg equilibrium for any of the collections (fig. 1*B*; supplementary table S2; Bonferroni-corrected χ^2^ test, *P *=* *1 for all). Thus, there was no evidence of the polymorphism being maintained by overdominant selection, which could result in an excess of heterozygotes.

**Table 1:** Effect of Sex and Season on Allele Frequency.

**Factor** ^a^	***Δ*** ^b^	*P*_CMH_	*P*_bootstrap_
Sex	0.06	0.0667	0.0208
Season F	0.041	0.0362	0.0639
Season M	−0.011	0.8043	0.8194

Note.—*P*_CMH_, *P*-value from CMH test; *P*_bootstrap_, *P*-value from bootstrapping test.

a Comparisons of observed cumulative difference in G frequency were performed between males (M) and females (F) across all collections (Sex) and between all June and all September collections for each sex (Season).

b Mean difference in G allele frequency across all collections between either males and females (F − M) or seasons (September − June) for each sex.

### Sexually Antagonistic and Temporally Fluctuating Selection Can Explain Allele and Genotype Frequency Dynamics at Position 67

In order to identify a plausible selection scenario that could explain the observed SNP frequency dynamics at position 67, we fit a model with viability selection to our observed data (fig. 1) assuming a “seasonal” environment in which a new “season” begins every year in June and in September and represents the interval between each pair of successive sampling points, resulting in a total of nine seasons in our data set, numbered 1–9 in chronological order. We then estimated selection parameters for males and females and female dominance parameters separately for each season using a nonlinear least squares approach, which for the viability selection model is equivalent to a maximum likelihood approach (supplementary text 1, Supplementary Material online). Fitness values were calculated relative to the C or CC genotypes, which were set to 1, for males and females, respectively (see supplementary text 1 and tables S4 and S5, Supplementary Material online).

**Fig. 2.**
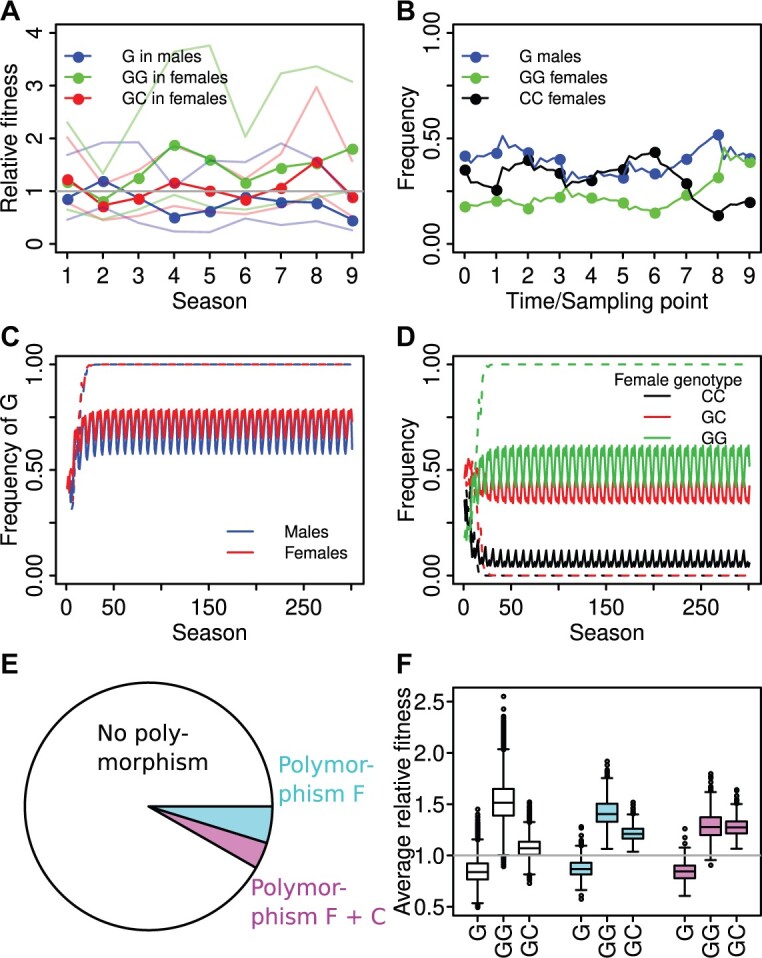
Modeling of variation at position 67. Because varying the number of generations per season resulted in a plateau of selection coefficients at five generations per season (supplementary text 1 and figs. S1 and S2, Supplementary Material online), all results are shown for five generations per season. (*A*) Estimates of relative fitness for G males (blue) relative to C males and for GG (green) and GC (red) females relative to CC females. The 95% likelihood profile confidence intervals are shown as light, solid lines. (*B*) Observed frequencies of the G allele in males (blue), and the GG (green) and CC (black) genotypes in females (points) and the corresponding predictions of the parameterized model (lines) starting at the observed frequencies for sampling point 1. Long-term projections of allele (*C*) and genotype (*D*) frequencies with model parameter estimates and using the season cycle 1, 2, 3, 4, 5, 6, 7, 8. The dashed lines represent projections with the estimated fitness values, whereas the solid lines represent projections with the dominance of the G allele in females set to 0.715, as estimated for starvation resistance in the Munich background. (*E*, *F*) Long-term predictions taking into account uncertainty in the parameter estimates. (*E*) The proportion of parameter sets for which polymorphism was not maintained (white; 91.7%), maintained only in the full model with temporal fluctuations (*F*; blue; 4.8%), or maintained in both the full model and the model with constant fitnesses set equal to the mean fitness of the respective genotype (F + C; pink; 3.5%) is shown. (*F*) The respective distributions of time-averaged fitness values for G males, GG females, and GC females are shown.

Our model predicts that, overall, selection was generally sexually antagonistic. Whenever the fitness of GG females was greater than 1 (i.e., they were favored), the relative fitness of G males was below 1, and vice versa (fig. 2*A* and supplementary fig. S1, Supplementary Material online). In most seasons, the G allele was beneficial in females and deleterious in males, but fitness fluctuated and in at least one season a reversal occurred (fig. 2*A* and supplementary fig. S1, Supplementary Material online), suggesting that temporal variation in selection may also be occurring. Our model also predicted nonmonotonic behavior of allele and genotype frequencies between sampling points (fig. 2*B* and supplementary fig. S2, Supplementary Material online). This observation is likely related to the sexually antagonistic selection we observed and to the fact that allele copies move back and forth between the male and female background. For example, all allele copies in males derive from allele copies in females in the previous generation. After the selection regime changes, it therefore takes one generation for selection effects in females to also affect male allele frequencies. Thus, the direction of allele frequency change often shifts after the first generation of the season. However, such effects may play a lesser role in more realistic settings with overlapping generations and a more gradual shift between seasons. The fitness of GC genotypes was generally close to 1, that is, the fitness of the CC genotype (fig. 2*A* and supplementary fig. S1, Supplementary Material online); thus, our model suggests that the G allele is fully or partially recessive in females. However, there were also exceptions where the fitness of GC females was as extreme or even more extreme than that of GG females (e.g., in seasons 1, 2, and 8; fig. 2*A* and supplementary fig. S1, Supplementary Material online), which suggests the occurrence of temporal changes in dominance in this population.

#### The Effect of Uncertainty in Allele Frequency on Parameter Estimates

Our model is based upon allele and genotype frequencies estimated from finite samples, which can be subject to sampling error. In order to determine how uncertainty in our allele and genotype frequency estimates translates into uncertainty in our parameter estimates, we computed confidence intervals for our parameter estimates in two ways: 1) We assumed that the observed frequencies are the true frequencies and sampled 1,000 new data sets assuming a binomial distribution for males and a multinomial distribution for females and estimated the parameters for each data set (supplementary text 1; supplementary figs. S4 and S8, Supplementary Material online); and 2) we computed likelihood profile confidence intervals (Bolker 2008) for our full model. Briefly, we used the probability mass functions of the binomial and multinomial distributions for males and females, respectively, to calculate the likelihood of a parameter combination, that is, the probability of obtaining the observed data set given the parameter combination, and then computed the likelihood profile for each of our fitness parameters (supplementary text 1, Supplementary Material online; fig. 2*A* and supplementary figs. S5–S8, Supplementary Material online). The 95% confidence intervals derived from these two independent approaches roughly agree (supplementary fig. S8, Supplementary Material online). Most confidence intervals overlap 1, indicating that there is little certainty about the direction of selection and dominance, with the exception of season 9, in which the confidence intervals of the relative fitness of G males and GG females do not overlap (fig. 2A), the relative fitness of G males was clearly below 1 according to both approaches, and the lower bound for GG females was close to 1 for the likelihood profile approach (fig. 2*A* and supplementary fig. S8, Supplementary Material online). Thus, when taking uncertainty surrounding our allele and genotype frequency estimates into account, we see clear indications that sexually antagonistic selection occurred during season 9 in which the G allele was male-detrimental but female-beneficial.

To better understand how our full model compares with simpler models when uncertainty is taken into account, we calculated Akaike’s information criterion (AIC). Models with lower AIC are considered to be better. A general rule of thumb is that models with differences in AIC (ΔAIC) less than 2 are equivalent, models with ΔAICs between 4 and 7 are clearly distinguishable; and models with ΔAICs greater than 10 are distinctly different (Bolker 2008); however, it should be noted that these cutoffs are rather arbitrary. We considered the full model as well as six simpler models representing neutrality and a range of selection scenarios with either temporally fluctuating or sexually antagonistic selection (supplementary text 1; supplementary table S6, Supplementary Material online). It should also be noted that AIC penalizes each additional parameter, and our full model contains more than twice as many parameters as the simpler models because it includes both temporally fluctuating and sexually antagonistic selection (30 vs. 14 in the next most complex model; supplementary table S6, Supplementary Material online). All the models with selection were better than neutrality (ΔAIC > 10 for all; supplementary table S6, Supplementary Material online). Based on AIC values, the best model is one with only fluctuating selection and G dominant in females (supplementary table S6, Supplementary Material online); however, this model is not clearly distinguishable from several of the other simple models, including one with fluctuating selection and G female-recessive (ΔAIC < 4; supplementary table S6, Supplementary Material online). Indeed, all the simpler models with selection that we considered fell somewhere on the spectrum between equivalent and clearly distinguishable (ΔAIC = 0.6–6.8, with the majority of ΔAIC ≤ 4.5; supplementary table S6, Supplementary Material online), suggesting that any of them might explain the data approximately equally well.

#### Sexually Antagonistic and Fluctuating Selection Can Maintain Polymorphism under Certain Conditions

In order to determine whether the observed dynamics and parameter estimates could maintain a stable polymorphism, we used our parameter estimates to iterate the dynamics for a large number of seasons by repeating a season cycle consisting of seasons 1–8, where season 9 was left out to balance the number of “summer” and “winter” seasons (choosing seasons 2–9 yields very similar results; see supplementary text 1 and figs. S11 and S12, Supplementary Material online). Our model with its estimated parameters predicted that the C allele will go extinct long term (fig. 2*C* and *D*, dashed lines; supplementary fig. S11, Supplementary Material online); however, this conclusion is sensitive to the estimated parameter values; for example, using a dominance coefficient of 0.715 instead (as observed for starvation resistance in the Munich background, see below) resulted in long-term stable polymorphism (fig. 2*C* and *D*, solid lines).

To determine the importance of sexually antagonistic versus temporally fluctuating selection in maintaining polymorphism, we also ran long-term predictions with all parameters constant over time and set to the average of their estimated values for the nine seasons (see supplementary text 1; supplementary table S7, Supplementary Material online). Again, in our model with the associated parameter estimates, the C allele is predicted to go extinct (supplementary fig. S13, Supplementary Material online) and thus our parameterized model with sexual antagonism alone cannot immediately explain the maintenance of polymorphism, likely because conditions for the maintenance of X-linked polymorphism by sexual antagonism are restrictive if the female-beneficial allele is recessive (Patten and Haig 2009). As in the variable case (fig. 2*C* and *D*), again coexistence was possible if the dominance coefficient *h* was set to 0.715 (supplementary text 1 and fig. S14, Supplementary Material online). Similarly, for the best model according to AIC with its estimated parameters, polymorphism was not maintained in the long run (supplementary fig. S15, Supplementary Material online).

Finally, we studied how uncertainty in the parameter estimates affects the long-term predictions (supplementary text 1, Supplementary Material online). For this, we randomly generated 20,000 parameter sets by sampling 24 fitness parameters (three genotype fitnesses for eight seasons, leaving out either season 1 or 9 as before to balance summer and winter seasons) in proportion to their profile likelihood (so that parameter values providing a better fit to the data are sampled more often). For each parameter set, we iterated the dynamics for 500 seasons, cycling repeatedly through the eight seasons. In 8.3% of parameter sets, polymorphism was maintained (fig. 2*E* and supplementary fig. S16, Supplementary Material online). For those parameter sets where polymorphism was maintained, we also performed simulations without temporal fluctuations and all fitness values set to their temporal average. Roughly 42% of those parameter combinations that had led to coexistence with fluctuations still allowed coexistence without fluctuations, whereas in the other cases polymorphism vanished. In the parameter sets that allowed for stable polymorphism both with and without fluctuations, the average fitness of GC females was similar to the average fitness of GG females (fig. 2*F* and supplementary fig. S16, Supplementary Material online). Thus, given the uncertainty in the sampled allele and genotype frequencies, our data are consistent both with a scenario in which polymorphism is maintained by sexually antagonistic selection with G beneficial and relatively dominant, and with scenarios where G is less dominant (i.e., approximately codominant), but fluctuating selection is necessary in addition to sexually antagonistic selection to maintain stable polymorphism at position 67. However, given that the majority of parameter sets did not lead to polymorphism, it is also plausible that additional selection patterns potentially playing out at other times or over different spatial scales are necessary to maintain polymorphism. Moreover, there might also be other models that are both consistent with the data and produce stable polymorphism with the estimated parameters that we did not consider here.

### Dominance at Position 67 Depends on Developmental Stage and Genetic Background

Our modeling results suggest that dominance at position 67 varies temporally, but if the G variant is favored in females, it is most often recessive; however, our estimates of dominance are subject to a high degree of uncertainty and our long-term modeling projections suggest that it is more likely to be dominant or codominant (supplementary text 1, Supplementary Material online). In order to better understand variation in dominance of the G allele at position 67 in natural populations, we calculated the degree of dominance for traits that *fiz* expression is known to affect. We did this in F2 offspring from reciprocal crosses between 1) two strains from a population in the Netherlands (henceforth NL), and 2) two strains from a population in Munich, Germany (henceforth MU). Briefly, for each genetic background, a derived GG and an ancestral CC variant isofemale strain from the same population were crossed to each other in both directions and F1 offspring were then mated with each other within each cross of each background. We then measured each trait in F2 females, which consist of heterozygous and homozygous individuals that have been reconstituted in a mixed genetic background of the two parental strains. We measured *fiz* expression, larval volume, adult body weight, wing length, wing area, and wing load index and calculated the degree of dominance, *h* (Falconer and Mackay 1996; Materials and Methods) of the derived G allele. Values of *h* between 0.5 and 1 represent partial to complete dominance and values between 0.5 and 0 represent partial to complete recessivity of the G allele. We then performed bootstrapping of our phenotypic measurements and reestimated *h* for each bootstrap replicate to obtain 95% confidence intervals for all examined traits. It should be noted that dominance estimates from our model and those we estimate from empirical data rely on different types of data and are unlikely to completely agree. Indeed, dominance based on allele frequency estimates represents dominance with respect to fitness, whereas dominance estimates based on phenotypic data reflect “phenotypic dominance.” However, empirical estimates should give us a general indication about patterns of dominance at position 67, which we can compare with predictions from our model.

#### Gene Expression

**Fig. 3.**
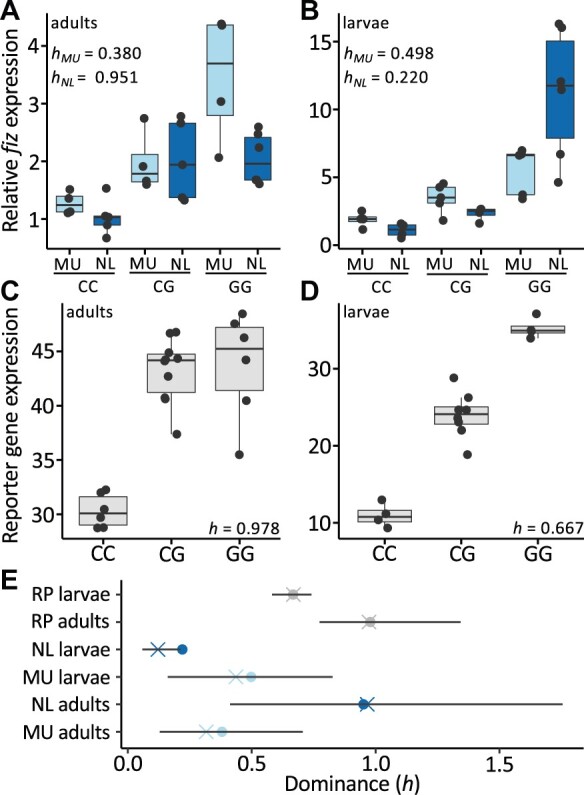
Dominance of variants at position 67 and gene expression. Relative *fiz* expression, as measured by qRT-PCR, in reconstituted F2 CC, CG, and GG female (*A*) adults and (*B*) larvae in the NL (dark blue) and MU (light blue) genetic backgrounds. Expression was calculated relative to the CC genotype in the NL background as 2^–(ΔCtX − ΔCtY)^, where ΔCtX is the mean ΔCt value for each sample of interest and ΔCtY is the mean ΔCt value of the NL CC genotype. β-Galactosidase reporter gene (RP) expression (gray) as measured spectrophotometrically in units of mOD/minute in CC, CG, and GG female (*C*) adults and (*D*) larvae. (*E*) Dominance (*h*; circles) of the G allele in females. The 95% confidence intervals and resampled mean (crosses) from 10,000 bootstrapping replicates are shown.

In keeping with previous studies using isofemale strains (Saminadin-Peter et al. 2012; Glaser-Schmitt et al. 2013; Glaser-Schmitt and Parsch 2018), *fiz* expression in reconstituted G homozygous females was 2- to 3.5-fold higher than in reconstituted C homozygous adults and 3- to 11-fold higher in larvae (fig. 3*A* and *B*). In adults, the G variant was estimated to be mostly dominant in the NL background but slightly recessive in the MU background; whereas, for larvae, it was moderately recessive in the NL background and approximately codominant in the MU background (fig. 3*A* and *B*). However, the confidence intervals overlapped for all pairs of our *h* estimates with the exception of NL larvae and adults (fig. 3*E*), suggesting that there are significant differences in the degree of dominance of the G allele between developmental stages in this background. In order to better understand the contribution of the variant at position 67 while minimizing potential background effects, we examined reporter gene expression in the same *trans*- background. Briefly, we measured β-galactosidase activity in transgenic strains containing the *fiz* enhancer with either a C or a G variant at position 67 upstream of a β-galactosidase gene (Glaser-Schmitt and Parsch 2018) as well as F1 hybrids of these two strains. In adults, estimated dominance most closely resembled that of the NL background, with the G allele almost completely dominant (fig. 3*C*). On the other hand, in larvae, the G allele was only partially dominant, which more closely resembled the MU background (fig. 3*D*). Based on nonoverlapping confidence intervals, there were significant differences in the degree of dominance between reporter gene adults and larvae as well as between the reporter gene strain and MU adults and NL larvae in the respective stages (fig. 3*E*). These results suggest that there are significant differences in the degree of dominance of the G allele between developmental stages and that variants at other loci in the natural populations modulate dominance at position 67 for *fiz* expression.

In order to better understand the influence of developmental stage and genetic background on variation and dominance of a single trait (i.e., gene expression), we first tested for an effect of the interaction between the allele at position 67 weighted by the estimated degree of dominance and developmental stage as well as the allele at position 67 weighted by the degree of dominance and genetic background on gene expression using an analysis of variance (ANOVA) utilizing all our gene expression data. We detected a significant effect of the interaction of both developmental stage and genetic background with genotype on gene expression (*P *=* *6.342 × 10^−7^ and *P *=* *0.0417, respectively), suggesting that within a single trait, there are significant differences in the effect of the allele at position 67 dependent upon the genetic background and developmental stage. It should be noted, however, that this test cannot distinguish between allelic effects and dominance. In order to test for differences in dominance, within each developmental stage and genetic background, we calculated individual dominance values for each heterozygote by rescaling heterozygote expression based on the means of the homozygotes for the respective stage and genetic background and tested for an effect of developmental stage and genetic background on dominance. We detected a significant effect of genetic background and the interaction of developmental stage and genetic background on dominance (*P *=* *0.0195 and *P *=* *0.0145, respectively; supplementary table S9, Supplementary Material online). Taken together, our results suggest that within a single trait, there is significant variation in the dominance of a genetic variant dependent upon the genetic background and developmental stage.

#### Body Size and Proportion Phenotypes

**Fig. 4.**
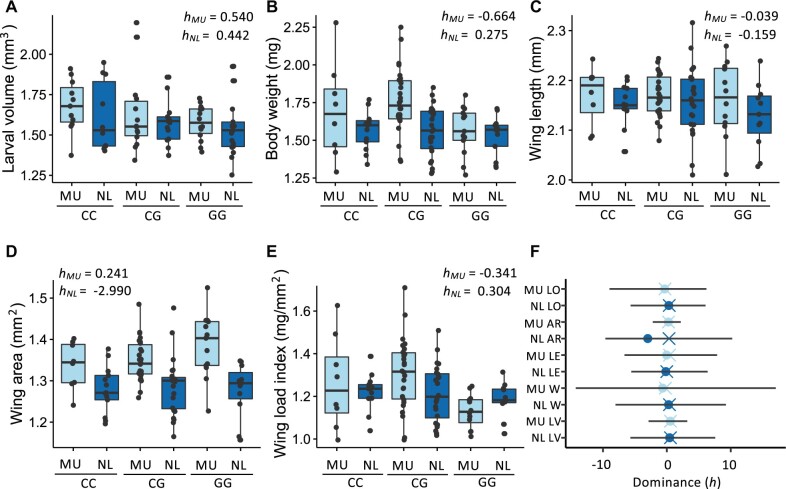
Dominance of variants at position 67 and body size and proportion. (*A*) Larval volume (LV), (*B*) body weight (W), (*C*) wing length (LE), (*D*) wing area (AR), and (*E*) wing load index (LO) in reconstituted F2 CC, CG, and GG females in the NL (dark blue) and MU (light blue) genetic backgrounds. (*F*) Dominance (*h*; circles) of the G allele in females. The 95% confidence intervals and resampled mean (crosses) calculated from 10,000 bootstrapping replicates are shown. (*B*–*E*) NL data taken from Glaser-Schmitt and Parsch (2018).

Expression of *fiz* and variation at position 67 have previously been shown to significantly affect larval growth, body size, and wing loading, a measure of proportional body size, with increased *fiz* expression associated with a decrease in all traits (Glaser-Schmitt and Parsch 2018). For all the examined traits, we estimated the G allele to be mostly recessive or, in one case, approximately codominant (fig. 4); however, the degree of dominance varied widely depending on both the genetic background and the trait under examination (fig. 4). Indeed, for all adult body size and wing loading traits, the heterozygote was more extreme than the C homozygote in at least one background (*h *<* *0), with the genetic background in which this occurred depending upon the trait under examination (fig. 4*B*–*E*). Moreover, these empirical estimates for phenotypic dominance are in line with those predicted by our model, namely that dominance of the G variant is mostly recessive but also variable. However, our confidence intervals were relatively large and overlapped between backgrounds and phenotype for all the examined traits (fig. 4*F*).

In order to assess the effect of the trait under examination and the genetic background on variation and dominance, we performed a meta-analysis utilizing data from all gene expression and body size-related traits. We first tested for an effect of the interaction between the allele at position 67 weighted by the estimated degree of dominance and the trait under examination as well as the allele at position 67 weighted by the degree of dominance and genetic background on the observed data using an ANOVA. We detected a significant effect of the interaction of both trait and genetic background with the genotype (*P *<* *10^−15^ and *P *=* *0.0140, respectively), suggesting that there are significant differences in the effect of the allele at position 67 dependent upon the genetic background and trait under examination. This test, however, cannot distinguish between allelic effects and dominance. In order to test for differences in dominance, within each developmental stage, trait, and genetic background, we calculated individual dominance values for each heterozygote as described above and tested for an effect of the examined trait and genetic background on dominance. We could not detect any significant differences in dominance (*P *>* *0.92 for all comparisons; supplementary table S9, Supplementary Material online). It should be noted, however, that this lack of significance does not necessarily imply that there is no effect of genetic background or the trait under consideration on dominance. Our heterozygote samples sizes are relatively small; therefore, it may be that we lack sufficient power to detect these differences with our data set.

### Variation at Position 67 Differentially Affects Starvation Resistance between the Sexes

**Fig. 5.**
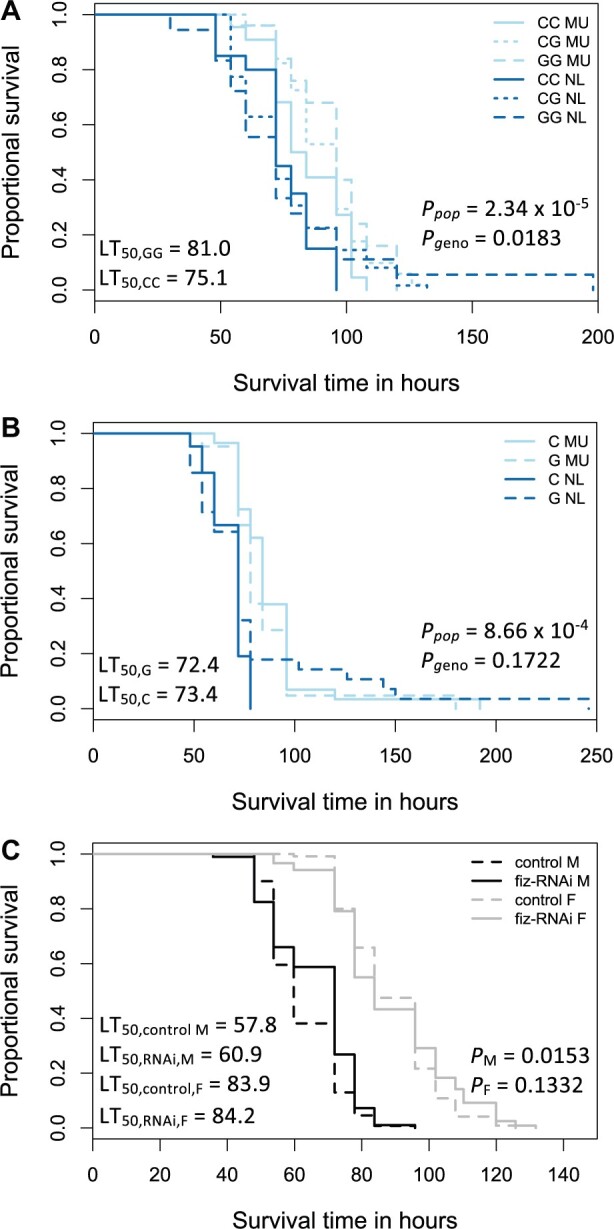
The effect of *fiz* expression on starvation resistance. Shown are survival curves under starvation conditions in reconstituted F2 (*A*) CC (solid lines), CG (dotted lines), and GG (hatched lines) females and (*B*) G (hatched lines) and C (solid lines) males in the NL (dark) and MU (light) genetic backgrounds and (*C*) in *fiz*-RNAi (solid lines) and control (hatched lines) males (M, dark) and females (F, light). Significance was assessed using a Cox proportional-hazards model with (*A*, *B*) genetic background, genotype assuming additivity, and vial as factors, or with (*C*) sex, line, and vial as factors. Median lethal time (LT_50_) for homozygotes, *fiz*-RNAi, and control strains are shown.

The effect of *fiz* expression and variation at position 67 on body size and proportion traits is concordant in both sexes (Glaser-Schmitt and Parsch 2018), suggesting that they are less likely candidates to be target(s) of differential selection between the sexes. In an examination of multiple phenotypic traits in inbred strains collected across multiple years and seasons from a single population in Turkey, only starvation resistance showed opposite reaction norms for males versus females, that is, female starvation resistance increased when male resistance decreased and vice versa (Önder BS, personal communication). Accordingly, we used RNAi to knock down *fiz* expression and determine whether it affects starvation resistance. Knocking down *fiz* expression significantly increased starvation resistance in males (Cox proportional-hazards model *P *=* *0.0153; fig. 5*C* and supplementary fig. S17*A*, Supplementary Material online), but not in females (Cox proportional-hazards model *P *=* *0.1332; fig. 5*C* and supplementary fig. S17*A*, Supplementary Material online). Indeed, when we calculated the median lethal time (LT_50_), that is, the time until 50% of individuals are dead, male LT_50_ increased by 5% when *fiz* expression was knocked down (60.9 vs. 57.8 h in the control line), whereas female LT_50_ changed by less than 0.05% (84.2 vs. 83.9 h in the control line).

In order to examine the effect of variation at position 67 on starvation tolerance, we performed crosses as described above and measured starvation resistance in reconstituted F2 homozygous and heterozygous females as well as hemizygous males. For females, we calculated the coefficient of dominance, *h*, and similar to expression and body size and proportion phenotypes (figs. 3 and 4), our dominance estimates varied depending on the genetic background, with the G allele partially dominant in the MU background (*h *=* *0.715; supplementary fig. S17*B*, Supplementary Material online) but the heterozygote slightly more extreme than the C homozygote in the NL background (*h* = -0.228; supplementary fig. S17*B*, Supplementary Material online); however, the confidence intervals for these *h* estimates were overlapping and relatively large (supplementary fig. S17, Supplementary Material online). The genetic background had a significant effect for both sexes (Cox proportional-hazards model; *P *<* *0.001 for both; fig. 5*A* and *B*). Similar to when *fiz* expression was knocked down, the sexes behaved differently depending on the variant at position 67. Homozygous G females survived 7.9% longer under starvation conditions than homozygous C females (LT_50_ of 81.0 vs. 75.1 h; fig. 5*A* and supplementary fig. S17*B*, Supplementary Material online); whereas, hemizygous G male survival time was reduced by 1.4% compared with hemizygous C males (LT_50_ of 72.4 vs. 73.4 h; fig. 5*B* and supplementary fig. S17*C*, Supplementary Material online). The effect of variation at position 67 was significant for females (*P *=* *0.0183; fig. 5*A* and supplementary fig. S17*B*, Supplementary Material online), but not for males (*P *=* *0.1772; fig. 5*B* and supplementary fig. S17*C*, Supplementary Material online). It may be that our detection of a significant effect in females but not in males is due to differences in statistical power. Indeed, for the observed effect sizes, our power to detect a significant effect in males was only 19%, whereas in females it was 77%.

Our detection of a significant effect of *fiz* genotype on starvation resistance in one sex but not the other in lab and wild-type backgrounds (fig. 5 and supplementary fig. S17, Supplementary Material online) suggests that there is an interaction between sex, *fiz* genotype, and starvation resistance. To test this, we performed a meta-analysis of starvation resistance in all tested backgrounds (MU, NL, *fiz*-RNAi, and RNAi control). Sex, *fiz* genotype, and genetic background all had a significant effect on starvation resistance (Cox proportional-hazards model *P *<* *0.0015 for all). Interestingly, we also detected a significant effect of the interaction between sex and *fiz* genotype (*P *=* *1.24 × 10^−12^), suggesting that sex is an important modulator of the effect of *fiz* genotype and the associated *fiz* expression level on starvation resistance. Although it is not strictly defined as a life-history trait, starvation resistance in *D*. *melanogaster* constitutes an important component of fitness because of its contribution to survival (reviewed in Flatt [2020]) and can be utilized as a reasonable proxy for fitness under assay conditions. However, because it is the combination of all fitness components that determines overall fitness and correlations often occur between these traits (reviewed in Flatt [2020]), further studies would be necessary to determine the relationship between starvation resistance and other fitness components that *fiz* expression affects. Taken together, our findings are in line with the sexually antagonistic selection predicted by our model if starvation resistance was the organismal trait under selection.

## Discussion

**Fig. 6.**
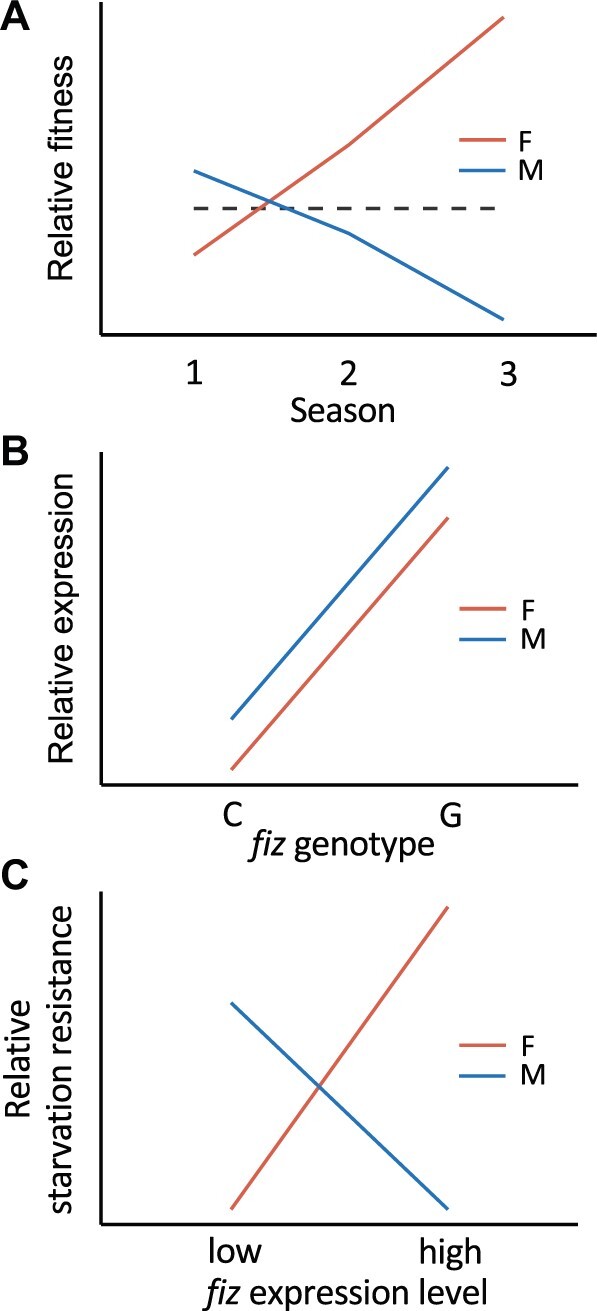
Proposed model of the effect of *fiz* genotype and expression level on fitness. (*A*) Selection at position 67 is sexually antagonistic and temporally fluctuating. Representative estimated fitness of GG females (F) and G males (M) in three selection phases (seasons) is shown. The dashed line represents the fitness of CC females or C males. Fitness values correspond to seasons 2–4 as depicted in figure 2*A*. (*B*) The G allele has a concordant effect on *fiz* expression level in males and females. Relative *fiz* expression within each sex is shown. (*C*) High *fiz* expression reduces starvation resistance in males but increases it in females. Relative starvation resistance within each sex is shown.

The G variant at *fiz* position 67 is currently at intermediate frequency in Europe (supplementary table S1, Supplementary Material online) and is present in isofemale strains collected approximately 30 years ago (Glaser-Schmitt et al. 2013; Glaser-Schmitt and Parsch 2018), suggesting that it has been maintained at intermediate frequency for at least several decades. Given that the G variant is present in all surveyed cosmopolitan populations, but absent in sub-Saharan Africa (Glaser-Schmitt and Parsch 2018), it likely has been segregating at considerable frequency since before *D. melanogaster’*s colonization of Europe, which occurred approximately 1,800 years ago (Sprengelmeyer et al. 2020). Indeed, it is possible that the polymorphism dates as far back as shortly after *D. melanogaster’*s expansion out of its ancestral range in sub-Saharan Africa, which occurred approximately 12,000 years ago (Sprengelmeyer et al. 2020). When we sampled a derived, European population (Munich, Germany) over a period of 5 years, we found a significant difference in allele frequency between the sexes as well as a significant change in allele frequency across seasons in females but not in males (fig. 1 and table 1). These empirical observations, along with our modeling results, suggest that a combination of sexually antagonistic and temporally fluctuating selection is acting on this SNP and may help maintain the G variant (figs. 2 and 6). However, it should be noted that we cannot rule out the possibility that another, unidentified scenario that we did not consider here is maintaining the observed polymorphism, such as strong interactions with other loci or polygenic adaptation (Jain and Stephan 2017; Barghi et al. 2019; Höllinger et al. 2019). Polygenic adaptation, however, is unlikely in this case because one of its hallmarks, nonparallelism between populations (Barghi et al. 2020), is absent. The point estimates for our model suggest that the G allele is female-beneficial and mostly recessive, although both the beneficial allele and dominance reversed in at least one season (fig. 2). However, there is a high degree of uncertainty in our estimates of selection and dominance for each season, with the exception of season 9 in which the G allele was clearly male-deleterious but female-beneficial (fig. 2*A* and supplementary fig. S8, Supplementary Material online). Therefore, the G allele is likely most often female-beneficial, but the dominance was less clear.

With the point estimates from our empirical data, our model does not predict that the combination of sexual antagonism and temporally fluctuating selection alone can maintain polymorphism at position 67 (fig. 2*C* and *D*); however, given the uncertainty in the parameter estimates, our data are also consistent with scenarios where the G allele is more dominant in females and where maintenance of polymorphism is then possible (supplementary text 1, Supplementary Material online; fig. 2*E* and *F* and supplementary fig. S16, Supplementary Material online). However, if the G allele is mostly recessive, additional patterns potentially playing out over different temporal or spatial scales are likely necessary. It may be that spatial variation in selection pressures and/or dominance play an important role in the maintenance of variation. For instance, there may be a geographic mosaic of dominance, where dominance changes depending on genetic background or local environmental conditions. Indeed, we found that dominance at position 67 is dependent on genetic background (fig. 3), suggesting that spatial variation in dominance driven by local genetic variation is a likely possibility and this spatial variation may help maintain polymorphism. It should also be noted that our model is based on only 5 years of observations and is deterministic with the assumption of an infinite population size. Nucleotide sequence polymorphism in the Munich population is comparable to that of other European populations (Kapun et al. 2020) and suggests the X chromosome has an effective population size (*N*_e_) greater than 1 million (Laurent et al. 2011). Although this *N*_e_ is relatively large, the population will be subject to genetic drift, which may lead to greater allele frequency fluctuations over time than predicted by the model.

In principle, patterns similar to those expected for sexually antagonistic viability selection could be caused by the G variant at position 67 having sex-specific effects on bait attraction, which would alter the allele frequencies between trapped males and females. To explain our results by bait preference alone, one would need to assume that the G allele increases bait attraction in females but decreases it in males. However, we sometimes see higher G frequency in males than in females (e.g., June 2016, 2017; fig. 1*A* and *D*). Furthermore, in June 2020 more than 50% of the males had the G allele (fig. 1*A*), which is a large increase over the preceding years. If the observed differences in allele frequency were caused solely by differences in bait attraction, then the male preference would have to have switched in this season so that the G allele led to greater attraction. For these reasons, we consider a bait effect to be unlikely to explain our observations.

In recent years, genome-wide population genetic approaches have emerged as useful tools in the identification and classification of putative sexual conflict; however, there are caveats to these approaches, making the identification of individual sexually antagonistic loci difficult (for a review see Mank [2017]). Therefore, identifying and overcoming challenges in the application of genomics to the study of sexual conflict has received much attention (Bissegger et al. 2020; Ruzicka et al. 2020). Moreover, the advent of these new approaches has fostered debate over the interpretation of oft-used earmarks of potential sex-specific selection, such as sex-biased gene expression (Cheng and Kirkpatrick 2016; Wright et al. 2018) and intersexual allele frequency differences (Cheng and Kirkpatrick 2016, 2020; Kasimatis et al. 2019; Mank et al. 2020). By genotyping individual flies for our target SNP, we were able to precisely determine the allele and genotype frequencies in each sex and season, which is not possible with genome-wide approaches such as pool-seq (Schlötterer et al. 2014). Additionally, our approach is not affected by cross-hybridization or misalignment of sex-chromosomal and autosomal sequences, which can lead to false inferences of sexual antagonism in sequencing and array-based studies (Bissegger et al. 2020; Kasimatis et al. 2021). A drawback, however, is that we lack information on how many other SNPs in the genome show similar frequency dynamics and if there are genome-wide demographic processes that might lead to false positives (Ruzicka et al. 2020). We can gain some insight into these issues from the DrosEU data (Kapun et al. 2020; Kapun et al. 2021), with the caveat that these data were generated by pool-seq of 40 males per season/location. The minor allele at *fiz* position 67 has a mean frequency of 37% across the DrosEU populations, which falls within the upper 1.22% of 25.9 million SNPs genome-wide and the upper 1.39% of 2.9 million X-linked SNPs. Thus, in terms of its frequency in European populations, the SNP at *fiz* position 67 is highly unusual, making it a strong candidate as a target of balancing selection. It is possible that sex-specific population structure, such as that caused by differences in migration rates between sexes, could produce a false signal of sexual antagonism (Ruzicka et al. 2020). However, this is unlikely to be the case for *D. melanogaster*, which shows little differentiation between European populations (mean *F*_st_ is 0.02 for the autosomes and 0.05 for the X chromosome) (Kapun et al. 2020).

Sexually antagonistic selection is thought to be of particular importance in the maintenance of polymorphism (Connallon and Clark 2014a, 2014b), but it behaves differently for sex chromosomes and autosomes because genes located on the X chromosome spend twice as much evolutionary time in females as in males. Theoretical studies have found that, on the X chromosome, polymorphism is more easily maintained the more recessive the male-beneficial allele is, or seen conversely, the more dominant the female-beneficial allele is (Patten and Haig 2009). In contrast, the more classic view of intragenomic conflict holds that the X chromosome should favor phenotypes closer to the female rather than the male optimum (Frank and Crespi 2011; Gardner and Úbeda 2017). However, a recent study suggests that these schools of thought are not as incongruent as previously thought (Hitchcock and Gardner 2020). Consistent with findings from theoretical studies, we found that polymorphism could be maintained long term when the G allele was more dominant (supplementary text 1, Supplementary Material online; fig. 2*E* and *F* and supplementary fig. S16, Supplementary Material online).

For our modeling parameters, the G allele was estimated to be mostly recessive with the dominance switching in several seasons, suggesting variation in dominance may be an important component shaping allele frequency dynamics at position 67; however, there was little certainty surrounding our dominance estimates. When we empirically estimated dominance for gene expression, body size and proportion, and starvation resistance traits, our results generally agreed with our model parameter estimates in that dominance of the G allele appeared to be mostly recessive, but varied dependent upon developmental stage, genetic background, and the trait considered (figs. 3 and 4 and supplementary fig. S17, Supplementary Material online); however, this variation was only significant within gene expression phenotypes. Similar to our modeling parameter estimates, the uncertainties surrounding many of our phenotypic dominance estimates were quite large (figs. 3 and 4 and supplementary fig. S17, Supplementary Material online), making it difficult to definitively estimate dominance for most examined traits, the exception being gene expression. However, within a single trait (gene expression), we were able to detect that degree of dominance significantly varies depending on the genetic background and development stage (fig. 3). Coupled with the significant interactions that we detected between the genotype at position 67 and the trait under examination and genetic background (supplementary table S8, Supplementary Material online), this variation in dominance suggests 1) that the relationship between *fiz* expression and phenotype is not necessarily linear and 2) that the interaction between variation at position 67 and genetic background influences the effect of *fiz* expression on final organismal phenotype. In other words, the effect of *fiz* regulatory variants on phenotype is dependent on the other genetic variants present. Consistent with this, a previous study found evidence for *trans*-acting factors affecting *fiz* expression segregating in natural populations (Glaser-Schmitt et al. 2018), which may affect both *fiz* expression and its effect on phenotype. Indeed, our results underscore that mutations affecting phenotype do not occur in a vacuum and the genetic background in which they occur can play an important role in the effect they have on the final organismal phenotype.

When we examined starvation resistance, we detected a significant interaction between sex, *fiz* genotype, and starvation resistance. We found that *fiz* expression and variation at position 67 both had a significant effect (fig. 5 and supplementary fig. S17, Supplementary Material online); however, these effects were highly sex-dependent (fig. 5 and supplementary fig. S17, Supplementary Material online). When *fiz* expression was knocked down in a lab strain, male starvation resistance increased but remained largely similar to the control for females (fig. 5*C* and supplementary fig. S17*A*, Supplementary Material online). On the other hand, the derived, high-expression G variant at position 67 in natural populations was associated with increased starvation resistance in females (fig. 5 and supplementary fig. S17, Supplementary Material online). Male starvation resistance slightly decreased, but this difference was not significant (fig. 5*B* and supplementary fig. S17*C*, Supplementary Material online); however, this lack of significance may be due to lower statistical power to detect small effects in males. Thus, in our assays we were only able to detect a significant effect of *fiz* expression on starvation resistance in males when *fiz* expression dropped from a very high level to very low, but only in females when *fiz* expression increased from an already high expression level. The *fiz* gene is normally constitutively expressed at very high levels in both males and females in the adult and larval Malpighian tubules and larval fat body (Leader et al. 2018) but shows male-biased expression (∼1.5-fold) in adult somatic tissues, including the Malpighian tubule and head (Gnad and Parsch 2006; Huylmans and Parsch 2014; Newell et al. 2016). This male-biased expression is conserved across *D. melanogaster* strains and in *D. simulans* (Gnad and Parsch 2006; Graze et al. 2014), suggesting that it predates the appearance of the SNP at position 67. Because *fiz* is located far away (18–36 kb) from the binding site of any dosage compensation complex component (Straub et al. 2013), it is unlikely that the mechanism of dosage compensation itself is responsible for its male biased expression, as has been proposed for some other X-linked genes (Huylmans and Parsch 2015; Belyi et al. 2020). Instead, it is likely to be the result of gene-specific regulation. A potential regulator is the male-specific protein product of the *fruitless* gene (Fru^M^), which has three binding sites within the *fiz* genomic region, including one within the *fiz* enhancer (Dalton et al. 2013).

It is possible that native *fiz* expression and its effect on starvation resistance are much higher than the male optimum so that a large decrease in *fiz* expression may have a significant effect on starvation resistance, but a comparatively small increase has a smaller effect, which we were unable to detect with our assay. Conversely, native *fiz* expression and its effect on starvation resistance may be lower than the female optimum so that an increase in *fiz* expression improves fitness, but a decrease has little effect. Therefore, it could be that native *fiz* expression and its effects on starvation resistance are at the edges of a relative fitness plateau for each sex so that changes in one direction have little effect but changes in the opposite direction have a larger effect (fig. 6). This selection may also be related to mating-associated sex differences, with increased starvation resistance being more important for females, who need additional resources for egg production. The sex-dependent effect of *fiz* expression and variation at position 67 on starvation resistance is in line with our model’s prediction of sexually antagonistic selection and suggests that it may be an organismal trait under selection. However, the mechanisms through which *fiz* expression affects starvation resistance remain unknown. Selection for increased adult starvation resistance has been shown to increase both body size and developmental time (Rion and Kawecki 2007; Hardy et al. 2018), which are two traits known to be affected by *fiz* expression (Glaser-Schmitt and Parsch 2018). The effect of *fiz* expression on adult starvation resistance may be related to its effects on these traits. A recent study revealed that there is a significant overlap between candidate genes involved in adaption to larval malnutrition and genes known to affect adult starvation resistance, although the relationship was antithetical (Kawecki et al. 2021). Indeed, the study found an enrichment of genes involved in hormonal signaling and metabolic processing among the candidates, including the downregulation of *fiz*, which has been shown to modulate active ecdysone levels (as measured by *E74B* expression; Glaser-Schmitt and Parsch 2018), in selected populations. Thus, it is possible that there are also evolutionary tradeoffs in nutritional stress between developmental stages as well as the sexes for *fiz* expression.

Previous studies have shown that seasonally fluctuating selection is able to maintain polymorphism in *Drosophila* populations and can help populations rapidly adapt to the changing seasons, producing a characteristic regular, cyclic pattern in allele frequency (Bergland et al. 2014; Behrman et al. 2018; but see Buffalo and Coop 2020). Although our model suggests that temporally fluctuating selection contributes to explaining the observed allele frequency dynamics at position 67, this temporal variation is not necessarily seasonal. Although the G allele frequency tended to be lower in June and higher in September, there were sometimes large fluctuations that did not correspond with the seasons, such as in 2018 and 2020 (fig. 1). Thus, the temporally fluctuating selection is unlikely to be purely seasonal. Because we also found that background has an effect on dominance of the G allele (fig. 3), these fluctuations may in part be the result of a shifting genetic background, with slight allele frequency shifts at other loci modulating dominance and therefore the selection coefficient at position 67. Another nonmutually exclusive possibility is that selection tends to fluctuate with the seasons but is also influenced by other factors that are unrelated to seasonality. This type of selection would also be consistent with selection for increased starvation resistance in females but decreased in males. Food availability likely fluctuates with the seasons but may also vary due to other factors, including climatic variables or anthropological factors such as agriculture or land development.

Although adult starvation resistance is the trait we found to be most consistent with temporally fluctuating, sexually antagonistic selection at position 67 (fig. 6), it is possible that a combination of this and/or other traits that we examined (or another trait that we have yet to identify as associated with *fiz* expression) are targets of selection. Indeed, our study underscores both the difficulty and the importance of characterizing individual cases of selection in natural populations. The identification of the organismal trait under selection is particularly difficult for genes with pleiotropic effects, especially if the phenotypes themselves are highly polygenic. However, characterizing these individual cases can improve our understanding of how selection occurs in natural populations, how this selection affects organismal phenotypes, and how it can influence levels of standing genetic variation.

## Materials and Methods

### *Drosophila melanogaster* Samples

All *D. melanogaster* strains and wild-caught flies were maintained at 21 °C with a 14 h light:10 h dark cycle on standard cornmeal–yeast–molasses medium unless otherwise stated.

#### Wild-caught Samples

Wild *D. melanogaster* were sampled from a population in Munich, Germany (latitude: 48.18, longitude: 11.61, altitude: 520) twice per year in late June and early September in 2016, 2017, 2018, 2019, and 2020, which corresponds to approximately the beginning and end of the breeding season in Munich. Sampling was performed at the same time each year and season, with 2.5 months (11–12 weeks) between the June and September collections and approximately 9.5 months between the September collection and June collection of the following year. Flies were collected using traps with apple-yeast bait and transferred to individual 35-ml vials containing standard medium. The species identity of all collected males was confirmed by visual inspection of the genitalia under a dissecting microscope in order to ensure that wild-caught flies were *D. melanogaster* rather than the closely related *D. simulans*. For each collection, a subset of the wild-caught females (50–90 females per collection) was allowed to lay eggs and species identity was confirmed from male offspring as described above. We did not detect any *D. simulans* in any of our collections. Collected flies were frozen individually and stored at −80 °C until DNA extraction. For the September 2019 collection, we tested for a deviation from a 50:50 sex ratio by counting male and female offspring for a minimum of 50 eclosed offspring from each of 40 wild-caught females.

#### Crosses to Test the Association of Variation at Position 67 with Phenotype

Larval volume, wing size, body weight, starvation resistance, and relative *fiz* expression were measured in the F2 offspring of two sets of reciprocal crosses between isofemale lines presenting either an ancestral C or a derived G at position 67. Reciprocal crosses of 30–40 females and 15–20 males were performed for each pair of isofemale lines, 40–50 F1 progeny were allowed to randomly mate, and phenotypes were measured in the F2 generation. Cross sets were performed using either two isofemale lines from Leiden, the Netherlands (NL01 and NL14) or two isofemale lines collected in June 2014 from the same Munich population as described above (MU06.14_17 and MU06.14_18). The Dutch lines were used in several previous studies about expression, phenotype, and/or sequence variation associated with the *fiz* enhancer (Saminadin-Peter et al. 2012; Glaser-Schmitt et al. 2013; Glaser-Schmitt and Parsch 2018).

#### *fiz* Knockdown Strains

A previous study on the effect of *fiz* expression on phenotype found highly congruous results when *fiz* expression was disrupted by a deletion in the coding region in a hypomorph strain and when expression was knocked down by RNAi (Glaser-Schmitt and Parsch 2018). Therefore, we tested for an effect of *fiz* expression on adult starvation resistance using a knockdown of *fiz* expression with an RNAi construct under the control of the yeast GAL4/UAS system. A *D. melanogaster* line producing a hairpin RNA complementary to *fiz* mRNA under the control of a UAS (ID: 107089) as well as a line containing an empty vector at the same genomic location (ID: 60100), which we used as a control, were obtained from the Vienna *Drosophila* Resource Center (Vienna, Austria) (Dietzl et al. 2007). These lines were crossed to an Act5C-GAL4/CyO driver line, and the progeny were used for adult starvation resistance assays. Using Real-Time Quantitative Reverse Transcription PCR (qRT-PCR), *fiz* expression knockdown efficiency for this RNAi and driver strain combination was previously estimated to be 98.6% for adult females and 98.9% for adult males (Glaser-Schmitt and Parsch 2018).

#### *fiz* Enhancer Reporter Gene Strains

Reporter gene strains containing *fiz* enhancer regions upstream of a *LacZ* reporter gene located at cytological band 86F on the third chromosome were generated and described in Glaser-Schmitt and Parsch (2018). Reporter gene expression was assayed in two reporter gene strains containing either a C or a G variant at position 67 in an otherwise identical cosmopolitan *fiz* enhancer as well as in the F1 hybrids of reciprocal crosses between these strains (see supplementary methods, Supplementary Material online).

### Phenotypic Measurements and SNP Genotyping of Position 67

For wild-caught flies and F2 flies and larvae, genotyping of the variant at position 67 was carried out using DNA extraction and PCR followed by a restriction enzyme-based assay (Glaser-Schmitt and Parsch 2018; see supplementary methods, Supplementary Material online). Larval volume, wing length, wing area, body weight, wing load index, and relative *fiz* expression were measured in F2 offspring in the NL and MU backgrounds (see supplementary methods, Supplementary Material online).

#### Starvation Assays

Starvation resistance was measured in F2 as well as *fiz* knockdown and control flies at 25 °C. Adult 5-day-old flies were placed individually, for F2 flies, or in groups of 20, for *fiz* knockdown and control flies, in small vials containing 1.5% agarose, which provides moisture but no nutrition, and mortality was recorded at regular intervals of every 6 or 12 h until all flies had died. F2 flies were tested individually rather than in groups because their genotype at the time of the assay is unknown. Upon their death, F2 flies were individually frozen for later genotyping. This handling difference should not affect starvation resistance measurements because 1) the number of individuals is low enough that flies could be quickly and easily scored, 2) the density of flies in each vial was low enough for each individual to have an abundance of freedom of movement before and after the death of its conspecifics, and 3) adults are unable to utilize their dead conspecifics as a food source without the presence of larvae to break them down (Vijendravarma et al. 2013). Starvation resistance was measured for 5–7 biological replicates per strain and sex for *fiz* knockdown and control flies or 18–62 individual flies per sex, genotype, and cross for F2 flies. Significance was assessed for each sex using a Cox proportional-hazards model (Therneau and Grambsch 2000) as implemented in the survival package (Therneau 2019) in R (R Core Team 2018) with genetic background, genotype assuming additivity (i.e., *h *=* *0.5; where CC = 0, GC = 1, GG = 2), and vial as factors for F2 flies, or with line and vial as factors for *fiz* knockdown and control flies. We further performed a meta-analysis of starvation resistance in all backgrounds (MU, NL, *fiz* knockdown and control) using a Cox proportional-hazards model with sex, strain/genetic background, *fiz* (CC/C, GC, GG/G, *fiz*-RNAi, control) genotype assuming additivity (where *fiz*-RNAi = 0, CC/C/control = 1, GC = 2, and G/GG =3), vial, and the interaction between sex and *fiz* genotype as factors. Control *fiz* expression was most similar to basal *fiz* expression in natural populations for both sexes (i.e., the CC/C genotype); therefore, control flies were treated as such in our approximation of additivity. For each sex and genotype or strain, LT_50_ was calculated from the total number of flies dead versus alive at each timepoint using the glm and dose.p functions as implemented in R (R Core Team 2018).

### Statistical Tests for Allele Frequency Differences

For our wild-caught samples, we tested for differences in allele frequency between seasons or sexes using both a CMH test and a boostrapping test. The advantage of both tests is that they use the exact counts of alleles observed in each sex, season, and year, which is important because the sample size varies in each of these categories for each collection. Further, the tests allow for the detection of consistent directional patterns across collections (e.g., consistently higher in one sex or season). The CMH test was applied to two-by-two tables of the G and C allele counts in the June and September collections within each sex across the 5 years, or in males and females across the ten collections. With the bootstrapping approach, we performed random binomial sampling of alleles within each sex and season. For this, we maintained the same sample size for each sex and season as in our observed data but randomly sampled alleles on the basis of their observed frequency in each year (for tests of seasons) or each collection (for tests of sex). For each test, we compared the observed cumulative difference in G frequency between June and September over all years (or between females and males over all collections) to those of 10,000 randomizations. The *P*-value was estimated as the proportion of randomizations with a cumulative difference greater than or equal to the observed value.

There are two main reasons why the above tests may give slightly different *P*-values. First, unlike the CMH test *P*-value, which is derived from a statistical distribution, the bootstrapping *P*-value is estimated by random resampling. Thus, the *P*-value will differ each time the test is run. For our data, we find that the run-to-run variation is typically around 1–2% when 10,000 replicates are performed. Second, the CMH test assumes that the allele frequencies in the population remain constant throughout the entire sampling period. This assumption could be violated if there is genetic drift and/or a consistent directional change in allele frequency. For example, if the frequency of the G allele increased monotonically from June 2016 through September 2020, then within each year the frequency would be higher in September than in June. Although this is not the case for our data (fig. 1*A*) and the CMH test appears to be relatively robust to genetic drift (Vlachos et al. 2019), it is difficult to intuit how deviations from this underlying assumption might influence the *P*-value. The bootstrapping approach gets around this limitation by allowing the overall allele frequency to vary among seasons (for the test of sexes) or among years (for the tests of seasons).

### Calculation of Degree of Dominance

For reconstituted F2 females in the MU and NL genetic backgrounds as well as homozygous and heterozygous reporter gene strains, we calculated the degree of dominance for all examined traits. Degree of dominance, *h*, was calculated as:

(1) $$h=\frac{X_{\mathrm{CG}}-X_{\mathrm{CC}}}{X_{\mathrm{GG}}-X_{\mathrm{CC}}},$$

where *X*_GG_, *X*_CC_, and *X*_CG_ represent the average phenotypic value of the GG, CC, and CG genotypes, respectively (Falconer and Mackay 1996). We log square root transformed the data for all traits, excepting qRT-PCR quantified gene expression and wing load index, which were square root transformed to avoid the generation of negative values, to improve the fit to normality and used the mean phenotype as the phenotypic value for each genotype. In order to estimate the uncertainty surrounding our estimates of *h*, we randomly resampled our phenotypic measurements with replacement for a total of 10,000 bootstrapping replicates. We then reestimated dominance for each bootstrap replicate and used these *h* estimate replicates to calculate 95% confidence intervals for dominance of the examined traits. In order to test for differences in dominance, for each trait, genetic background, and developmental stage, we calculated individual dominance values for each heterozygote by rescaling each heterozygote value as in equation (1) using the corresponding mean homozygote values. We then tested for an effect of genetic background, developmental stage, and/or trait under examination as well as any interactions on dominance with an ANOVA. It should be noted that although only heterozygotes are used in this test, the homozygotes play an important role in the rescaling. Significance of the effect of the interaction of the allele at position 67 with genetic background and the trait examined was assessed with an ANOVA using data from all gene expression and body size-related traits with allele, trait, genetic background, the interaction of trait and allele, and the interaction of genetic background and allele as factors. We similarly tested for significance of the effect of the interaction of the allele at position 67 with genetic background and the developmental stage within a single trait (i.e., gene expression) using all gene expression data. In these analyses, homozygotes were weighted as 0 (CC) and 1 (GG) and heterozygotes were weighted by our estimated degree of dominance for the respective background and trait or stage. To ensure that using degree of dominance estimated from our data in our analyses did not introduce bias in the results, we repeated these analyses using the mode of dominance with the dominance for each trait and population categorized into dominance classes (recessive, partially recessive, codominant, partially dominant, dominant, and heterozygote more extreme than either homozygote; where *h* = 0, 0.25, 0.5, 0.75, 1, and −1, respectively) as well as with dominance categorized according to the types above, but without any assumptions of how each type of dominance affects the data using categorical variables. The results were concordant with results utilizing our estimates of *h* (supplementary table S8, Supplementary Material online). We, therefore, focus on the analyses utilizing our estimated degree of dominance in the main text.

### Modeling

In order to better understand the selective forces acting on and the mechanisms maintaining variation at position 67, we fit a population genetic model to our wild-caught data. We initially considered two models, one with viability selection and another with fecundity selection (see supplementary text 1, Supplementary Material online, for details). Both models predicted that selection was generally sexually antagonistic and exhibited nonmonotonic behavior of allele frequencies between sampling points (supplementary text 1 and fig. S2, Supplementary Material online); however, viability selection better fit our empirical data (see supplementary text 1, Supplementary Material online, Supplementary Material online), therefore we have focused on this model in the main text. The model assumed that selection acts at a single locus on the X chromosome with two alleles, and no new mutations at the time scale under consideration. Generations are discrete and the population size is constant and large enough such that genetic drift can be neglected (deterministic model). Further, we assume a 50:50 sex ratio, promiscuous mating, and random union of gametes. The environment is seasonal with a new season beginning every year in June and in September and spanning the interval between collections. Selection pressures acting at the locus can vary between males and females and between seasons. To estimate the selection and dominance parameters for each season, we fit the model to the observed data separately for each of the nine seasons (interval between successive sampling points) in the data set and used the optim function in R (R Core Team 2018) with method “L-BFGS-B” to find the parameter combination minimizing the sum of squared relative differences between observed frequencies and predicted frequencies at the end of the season. For our parameter estimates, we then iterated the dynamics for a large number of seasons to determine whether polymorphism is maintained long term (see supplementary text 1, Supplementary Material online). To estimate uncertainty in our parameter estimates, we calculated likelihood profile confidence intervals as well as confidence intervals from 1,000 simulated data sets assuming a binomial distribution for males and a multinomial distribution for females (see supplementary text 1, Supplementary Material online).

## Supplementary Material

Supplementary data are available at *Molecular Biology and Evolution* online.

## Supplementary Material

msab215_Supplementary_Data
