# Supplementary material for: Sexual Antagonism, Temporally Fluctuating Selection, and Variable Dominance Affect a Regulatory Polymorphism in Drosophila melanogaster
Source: Mol Biol Evol. 2021 Jul 21;38(11):4891–907. doi: 10.1093/molbev/msab215 (PMC8557461; doi:10.1093/molbev/msab215)
Supplement: msab215_Supplementary_Data [file msab215_supplementary_data.zip › Fig_S17.pdf]

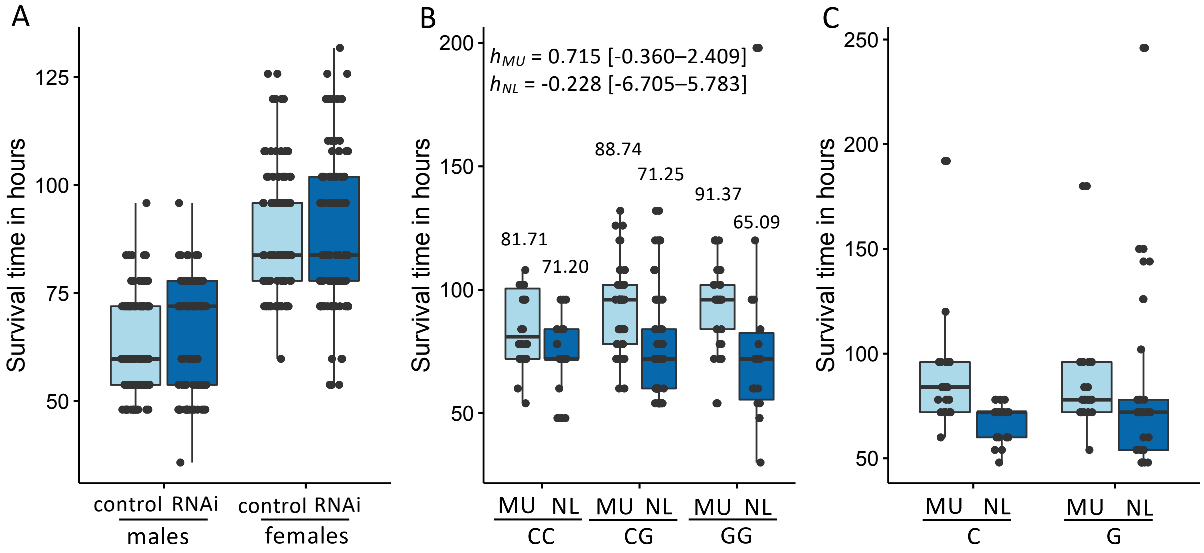


**Supplementary Figure S17: Dominance of variants at position 67 and starvation resistance.**

Survival time under starvation conditions in (A) *fiz*-RNAi (dark blue) and control (light blue) males and females, and in reconstituted F2 (B) CC, CG, and GG females and (C) G and C males in the NL (dark blue) and MU (light blue) genetic backgrounds. (B) Degree of dominance, *h*, for variants at position 67, calculated in comparison to the GG genotype using log square root transformed data, is shown. 95% confidence intervals from 10,000 bootstrap replicates are shown. Harmonic means for each genotype in each population are shown above the respective boxplot.
