# Supplementary material for: Sexual Antagonism, Temporally Fluctuating Selection, and Variable Dominance Affect a Regulatory Polymorphism in Drosophila melanogaster
Source: Mol Biol Evol. 2021 Jul 21;38(11):4891–907. doi: 10.1093/molbev/msab215 (PMC8557461; doi:10.1093/molbev/msab215)
Supplement: msab215_Supplementary_Data [file msab215_supplementary_data.zip › SuppText1.pdf]

Sexual antagonism, temporally fluctuating selection, and variable dominance affect a regulatory polymorphism in *Drosophila melanogaster*
Supplementary Text
Model description and results

Amanda Glaser-Schmitt, Meike J. Wittmann, Timothy J. S. Ramnarine & John Parsch

June 23, 2021

Goal

The goal is to find a plausible selection scenario explaining the observed dynamics at the SNP 67 G/C polymorphism in Munich wild-caught flies (Fig. 1) and shed light on potential mechanisms for the maintenance of polymorphism at this site.

Model

General assumptions

We model a population with discrete non-overlapping generations. The population size is constant and large enough so that we can neglect genetic drift and allele frequency dynamics can be described by a deterministic model. Selection acts at a single locus located at the X chromosome such that males have one copy and females two copies. There are two alleles, C and G and we assume that there are no new mutations at the time scale under consideration. We further assume a 50:50 sex ratio and promiscuous mating with random union of gametes.

The environment is seasonal and a new season starts every year in June (for the summer season) and in September (for the winter season) at the respective sampling points. We number the seasons as follows: season 1 is from June 2016 to September 2016, season 2 from September 2016 to June 2017, season 3 from June 2017 to September 2017, and so on. There are g generations per season (different numbers for summer vs. winter are possible). Selection pressures acting at the locus can vary between males and females and between seasons as given by Tables S4 and S5. Since population size and sex ratio are constant, only relative fitnesses matter and we take all fitness values relative to the C or CC types.

We consider two model versions that differ in the order of events in the life cycle.

Table S4 Fitness values of the two possible male genotypes in season i

Genotype	Fitness
C	1
G	$w_{m,i}$

Table S5 Fitness values of the three possible female genotypes in season i

Genotype	Fitness
CC	1
GC	$w_{f,GC,i}$
GG	$w_{f,GG,i}$

Model version with fecundity selection

In this model version, we assume the following life cycle, i.e. order of events in each generation:

1. Adults contribute to the egg and sperm pool in proportion to their fitness.
2. Males in the next generation are generated by drawing one random allele copy from the egg pool.
3. Females in the next generation are generated by independently drawing one allele from the egg pool and one allele from the sperm pool.
4. Census

We now consider the allele-frequency and genotype-frequency dynamics over one season, i. We keep track of the frequency of the G allele in males ($p_{m,t}$) and of the frequencies of the two homozygous genotypes in females ($p_{CC,t}$ and $p_{GG,t}$) in generation t of the season. The frequencies of the C allele in males and the frequency of heterozygotes in females follow directly from these quantities. The initial conditions, i.e. the frequencies before the start of the season, are $p_{m,0}$, $p_{CC,0}, p_{GG,0}$.

The frequencies of the G allele in the egg pool and sperm pool in generation t are

$$p_{\text{egg},t} = \frac{p_{GC,t}/2 \cdot w_{f,GC,i} + p_{GG,t} \cdot w_{f,GG,i}}{\bar{w}_{f,t}} \tag{S1}$$

and

$$p_{\text{sperm},t} = \frac{p_{m,t} \cdot w_{m,i}}{\bar{w}_{m,t}}, \tag{S2}$$

where

$$\bar{w}_{f,t} = p_{CC,t} + p_{GC,t} \cdot w_{f,GC,i} + p_{GG,t} \cdot w_{f,GG,i}. \tag{S3}$$

is the average fitness of females in generation t of season i and

$$\bar{w}_{m,t} = 1 - p_{m,t} + p_{m,t} \cdot w_{m,i} \tag{S4}$$

is the average fitness of males.

The allele and genotype frequencies in the next generation are then given by

$$p_{m,t+1} = p_{\text{egg},t}, \tag{S5}$$

$$p_{CC,t+1} = (1 - p_{\text{sperm},t})(1 - p_{\text{egg},t}), \tag{S6}$$

$$p_{GG,t+1} = p_{\text{sperm},t} \cdot p_{\text{egg},t}, \tag{S7}$$

and

$$p_{GC,t+1} = 1 - p_{CC,t+1} - p_{GG,t+1}. \tag{S8}$$

Model version with viability selection

In this model version, we assume the following life cycle, i.e. order of events in each generation:

1. All adults contribute equally to the egg and sperm pool.
2. Male offspring are generated by drawing one random allele copy from the egg pool.
3. Female offspring are generated by independently drawing one allele from the egg pool and one allele from the sperm pool.
4. Viability selection acts.
5. Census

Given allele/genotype frequencies $p_{m,t}$, $p_{CC,t}$ and $p_{GG,t}$ at the end of generation t, the G allele frequencies in the egg and sperm pool are:

$$p_{\text{egg},t} = p_{GC,t}/2 + p_{GG,t} \tag{S9}$$

and

$$p_{\text{sperm},t} = p_{m,t}. \tag{S10}$$

Before selection, the G allele frequency among male offspring is then

$$p_m^* = p_{\text{egg},t} \tag{S11}$$

and the frequencies of the CC and GG genotypes among female offspring are

$$p_{CC}^* = (1 - p_{\text{egg},t}) \cdot (1 - p_{\text{sperm},t}) \tag{S12}$$

and

$$p_{GG}^* = p_{\text{egg},t} \cdot p_{\text{sperm},t}. \tag{S13}$$

The frequencies at the end of generation $t+1$, i.e. among adults after selection, are

$$p_{m,t+1} = \frac{p_m^* \cdot w_{m,i}}{\bar{w}_{m,t}}, \tag{S14}$$

where

$$\bar{w}_{m,t} = p_m^* \cdot w_{m,i} + (1 - p_m^*), \tag{S15}$$

and

$$p_{CC,t+1} = \frac{p^*_{CC}}{\bar{w}_{f,t}} \tag{S16}$$

and

$$p_{GG,t+1} = \frac{p^*_{GG} \cdot w_{f,GG,i}}{\bar{w}_{f,t}}, \tag{S17}$$

where

$$\bar{w}_{f,t} = p^*_{CC} + p^*_{GG} \cdot w_{f,GG,i} + (1 - p^*_{CC} - p^*_{GG}) \cdot w_{f,GC,i}. \tag{S18}$$

Parameter estimation

We fit the model to the observed data (see Fig. 1) separately for each of the nine seasons (interval between successive sampling points) in the data set. We used the `optim` function in R with method "L-BFGS-B" to find the parameter combination minimizing the sum of squared relative differences (ssrd) between observed frequencies and predicted frequencies at the end of the season. All three fitness values were restricted to be between 0 and 10. The starting parameter guesses in each case were $w_{m,i} = w_{f,GC,i} = w_{f,GG,i} = 1$, which corresponds to neutrality.

Fig. S1 shows parameter estimates for each season under the fecundity or viability selection model with 2, 5, or 10 generations per season. See supplementary spreadsheet for all parameter estimates and the corresponding ssrd values. For all seasons, the viability-selection model gave lower ssrd values than the fecundity-selection model, suggesting that the viability-selection model fits better to the observations.

Both model versions agree in that selection is generally sexually antagonistic. That is, whenever the fitness of G males was below 1, the fitness of GG females was above 1, and vice versa. Interestingly though, the two model versions estimated almost the opposite temporal patterns of fitness values. For example, under fecundity selection, the G allele was favored in males in all seasons but season 2, whereas under viability selection it was only favored in season 2. These opposite results could be explained by the reversed order of selection and census in the two model versions.

For most seasons, the fitness of the GC genotype in females is closer to one than the fitness of the GG genotypes, indicating that the G allele is recessive. However, there are also exceptions where the fitness of GC females is as extreme or even more extreme than that of GG females (this was the case especially in the first two seasons in the data set). These cases hint at temporal changes in dominance.

Comparing the estimated fitness values for different numbers of generations per season (compare the different rows in Fig. S1) shows that selection coefficients (deviations from one) become smaller as the number of generations is increased from 2 to 5 (first vs. second row), but then stay roughly constant as the number of generations is further increased to 10 (second vs. third row). The general direction of the effect makes sense since to achieve the same change in allele frequencies, selection can be weaker per generation if it has more generations to play out.

Fig. S2 shows observed frequencies (points) together with model predictions (lines). For viability selection (right column), the model fit is essentially perfect, whereas for fecundity selection (left column), there are some deviations. The model predictions are also plotted for the unobserved generations between the sampling points. Surprisingly, both model versions predict non-monotonic

behavior of allele frequencies between sampling points. Allele and genotype frequencies often change very strongly in the first generation of a season, i.e. after the selection regime has changed, and then change more slowly or even start going back to the original values. This appears to be related to the sexually antagonistic selection and to the fact that allele copies move back and forth between the male and female background. For example, if the G allele is advantageous in males, but disadvantageous in females, G males might produce more offspring, but the males in the next generation receive all their alleles from females and there the C allele is favored.

We also attempted to fit the data with just two sets of parameters, one for summer (June to September) and one for winter (September to June), but this did not produce satisfactory fits.

The reason why the viability-selection model gives a perfect fit to the data, but the fecundity-selection model does not, is related to the differences in life cycle. In the viability-selection model, selection acts between random union of gametes and sampling. Thus, for any combination of observed frequencies, selection coefficients can be chosen to give these values. In the fecundity-selection model, individuals reproduce according to their fitnesses and then there is random union of gametes, followed by sampling. Now consider a season with male allele and genotype frequencies p_m, p_{CC}, and p_{GG} at the end. From (S5), we can infer that the frequency of the G allele in eggs just before was p_m. And from (S7), we can then infer the G allele frequency in sperm as p_{GG}/p_m. According to (S6), the frequency of the CC genotype would then be $(1-p_{\text{sperm}})(1-p_{\text{egg}})$, but this will generally not match the observed value. In fact, the observed values of the CC genotype are in most seasons larger than the expected ones based on this calculation (Fig. S3). This suggests that a pure fecundity-selection model is not consistent with the data.

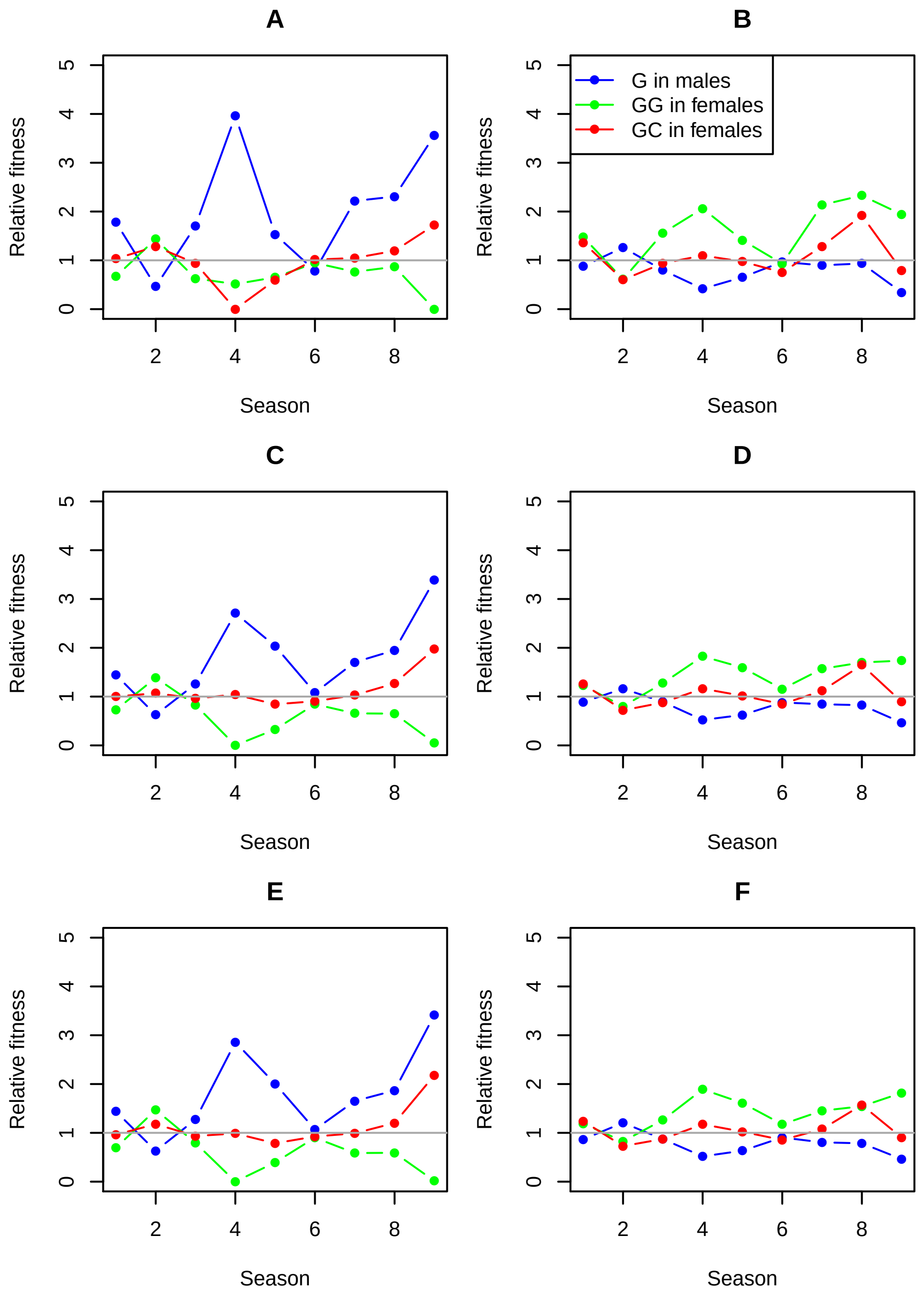

Figure S1 Estimates of relative fitness for G males (relative to C males), and for GG and GC females (relative to CC females. The first column (A, C, E) assumes fecundity selection and the second column (B, D, F) viability selection. The first row (A, B) assumes 2 generations per season, the second row (C, D) 5, and the third row (E, F) 10.

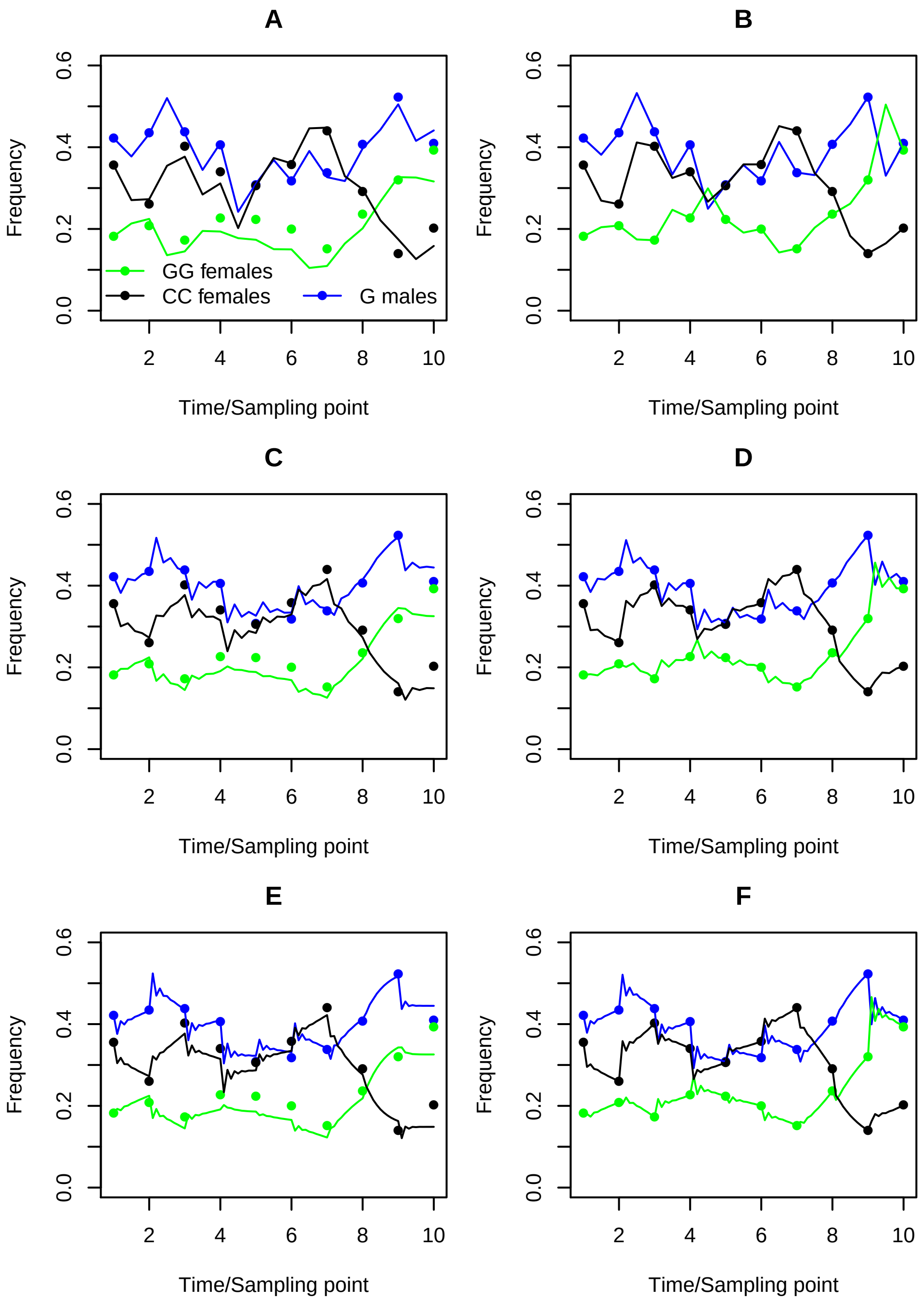

Figure S2 Observed frequencies of the G allele in males, and the GG and CC genotypes in females (points) and the corresponding predictions of the parameterized models (lines) starting at the observed frequencies for sampling point 1. The first column (A, C, E) assumes fecundity selection and the second column (B, D, F) viability selection. The first row (A, B) assumes 2 generations per season, the second row (C, D) 5, and the third row (E, F) 10. Model predictions for intermediate generations are also shown.

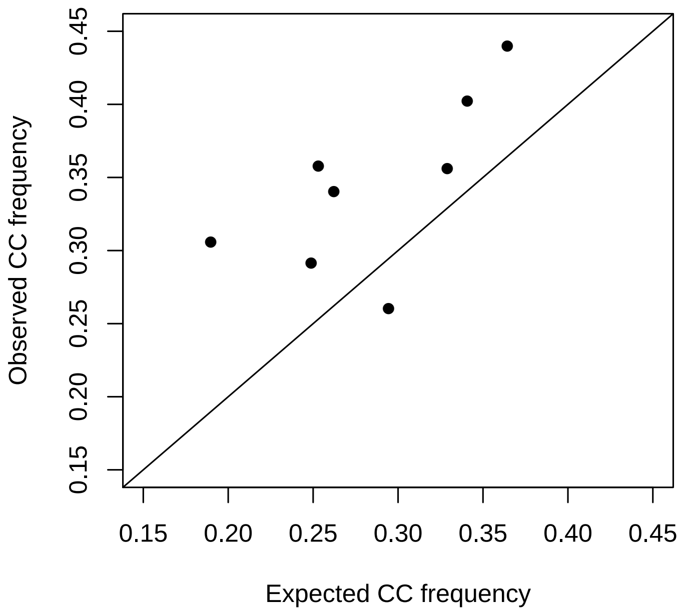

Figure S3 Observed frequency of the CC genotype vs. expected frequency in the fecundity-selection model.

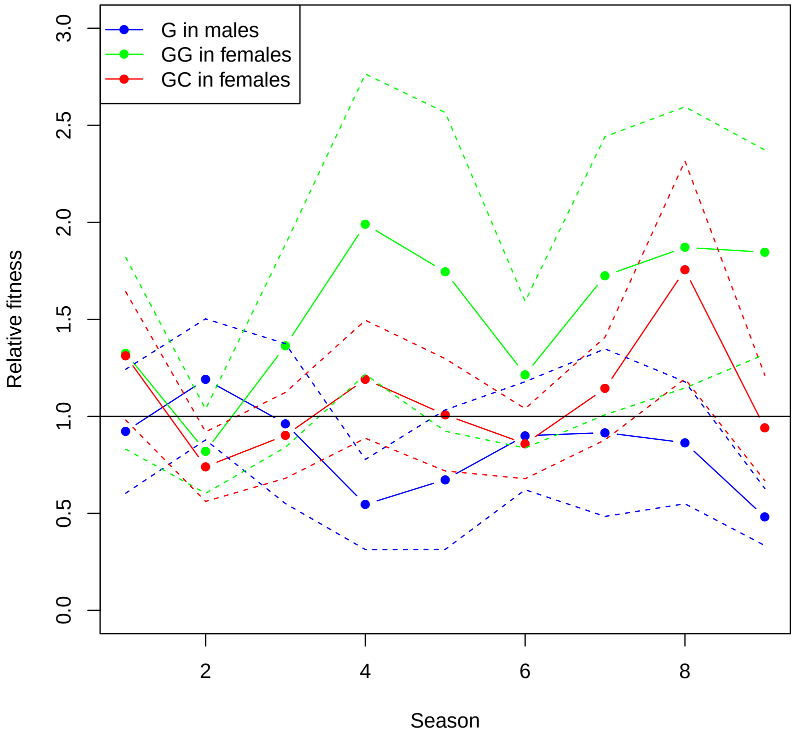

Figure S4 Mean fitness estimates ± standard deviation across simulated data sets assuming that the observed allele and genotype frequencies are the true ones. Based on the viability selection model with 5 generations per season.

Uncertainty in parameter estimates

The allele and genotype frequencies that underlie the estimated fitness values are based on samples of finite size and therefore subject to sampling error. Thus we determined how the uncertainty in estimates of allele and genotype frequencies translates into uncertainty in the parameter estimates. In these analyses, we focus on the viability selection model with five generations per season.

As a first approach, we assumed that the observed frequencies are the true frequencies and sampled 1000 new data sets with the same sample sizes for males and females at each time point as in the real data. Here we assumed that the number of G males follows a binomial distribution and the number of females of each genotype follows a multinomial distribution. For each simulated data set, we estimated the parameters as described above. The mean (± standard deviation) of the fitness estimates across the 1000 simulated data sets is shown in Fig. S4.

As a second approach, we computed likelihood profile confidence intervals (as described in Bolker 2008, Chapter 6). Our full model has 30 parameters, 27 fitness parameters: $w_{m,i}, w_{f,GC,i}$, and $w_{f,GG,i}$ for $i = 1, \ldots, 9$, plus the initial allele and genotype frequencies $p_{m,0}, p_{GG,0}$, and $p_{CC,0}$ (p_{GC} then automatically follows), which are also not known exactly because of sampling error. Given a parameter combination we then used the viability selection model described above to

compute the allele and genotype frequencies at the later sampling points $p_{m,i}, p_{GG,i}$, and $p_{CC,i}$ for $i = 1, \ldots, 9$.

Let $N_{m,i}$ and $N_{f,i}$ be the sample sizes of males and females at time point $i = 0, \ldots, 9$. Let $n_{m,i}$ be the number of G males in sample i, and $n_{GG,i}$, $n_{GC,i}$, and $n_{CC,i}$ the female genotype counts in sample i. Using the probability mass functions of the binomial distribution for the male samples and the multinomial distribution for the female samples, the likelihood of a parameter combination, i.e. the probability of obtaining the observed data set given the parameter combination is

$$L = \prod_{i=0}^{9} \binom{N_{m,i}}{n_{m,i}} \cdot p_{m,i}^{n_{m,i}} \cdot (1 - p_{m,i})^{N_{m,i} - n_{m,i}} \cdot \frac{N_{f,i}!}{n_{GG,i}! n_{GC,i}! n_{CC,i}!} \cdot p_{GG,i}^{n_{GG,i}} \cdot p_{GC,i}^{n_{GC,i}} \cdot p_{CC,i}^{n_{CC,i}}. \tag{S19}$$

Note that although we initially estimated the parameters separately for each season using nonlinear least squares, it turns out that the estimated fitness values for the viability selection model are equal to the maximum likelihood estimates for the fitness values in the full model. This is because for the viability selection model, the dynamics with the estimated parameters exactly matches the observed allele and genotype frequency dynamics and for the binomial and multinomial model, the probability of obtaining a certain sample frequency is maximized if the probability matches the sample frequency.

We then computed the likelihood profile for each of the 27 fitness parameters. For this, we fixed the focal parameter at a range of values while the respective other 29 parameters were tuned to minimize the negative log-likelihood, again using the `optim` function in R with method "L-BFGS-B". Here we used the least squares parameter estimates as starting values for the optimization. The initial frequency parameters were bounded between 0.1 and 0.9, and the fitness parameters were bounded between $e^{-1.5} = 0.22$ and $e^{1.5} = 4.5$. The results are shown in Figs. S5–S7. The boundaries of the 95% confidence intervals are given by the cutoff value for the negative log-likelihood, $-\log \hat{L} + \frac{\chi_1^2(\alpha)}{2}$, where $\hat{L}$ is the maximum likelihood and χ_1^2 is the is the quantile function of the χ^2-distribution with one degree of freedom.

In Fig. S8 we plot the parameter estimates and likelihood profile confidence intervals together with the resampling mean parameter estimates $\pm$ two standard deviations. The intervals derived from the two independent approaches roughly agree. Most confidence values overlap 1, indicating that there is little certainty about the direction of selection. An exception was season 9, where the relative fitness of G males was clearly below 1. Also for GG females, the likelihood profile confidence interval did not overlap 1, although the lower boundary was close to 1.

Finally, we performed model choice based on Akaike's information criterion (AIC). In addition to the full model with its 30 parameters, we considered a number of simplified candidate models (Table S6). In the model without temporally fluctuating selection, the relative fitness values of G males, GG females, and GC females were constant over time, such that it had 6 parameters (3 initial frequency parameters plus 3 fitness parameters). In the model with only fluctuating selection with G dominant, $w_{m,i} = w_{GG,i} = w_{GC,i}$ for all seasons, such that the model had 9 parameters plus 3 initial fitness parameters. In the model with only fluctuating selection with G recessive, $w_{m,i} = w_{GG,i}$ and $w_{GC,i} = 1$ for all seasons, giving rise to 12 parameters as well. In the fluctuating selection model with fixed factors, $w_{m,i} = f_m \cdot w_{GG,i}$ and $w_{GC,i} = f_h \cdot w_{GG,i}$, i.e. the fitness values of G males and of heterozygotes were linked via constant factors to the fitness of GG females for each time. This model had 14 parameters (3 initial frequencies + 9 values for the fitness of GG

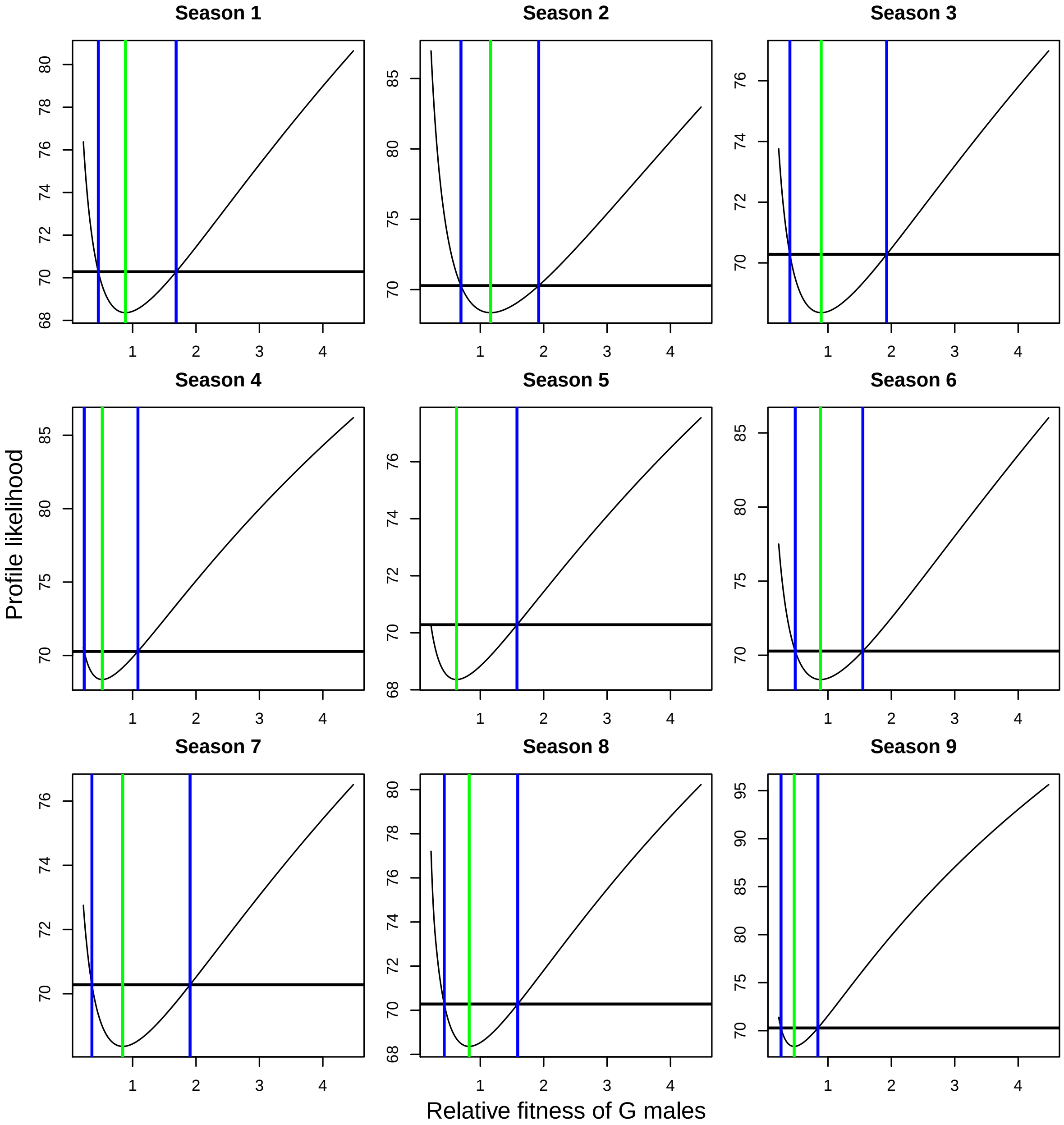

Figure S5 Likelihood profiles for the fitness of G males in each season, $w_{m,i}$. The value on the y-axis is the minimum negative log-likelihood with the focal parameter fixed at the value indicated on the x-axis. The green vertical lines indicate the least squares estimate, which is equal to the maximum likelihood estimate. The horizontal black line indicates the cutoff value for the confidence interval and the vertical blue lines indicate the upper and lower boundaries of the profile likelihood confidence intervals. For season 5, the negative log-likelihood for the left-most point is just below the cutoff value $e^{-1.5} = 0.22$. In Fig. 2 in the main text, the lower boundary of the confidence interval was set to this maximal value.

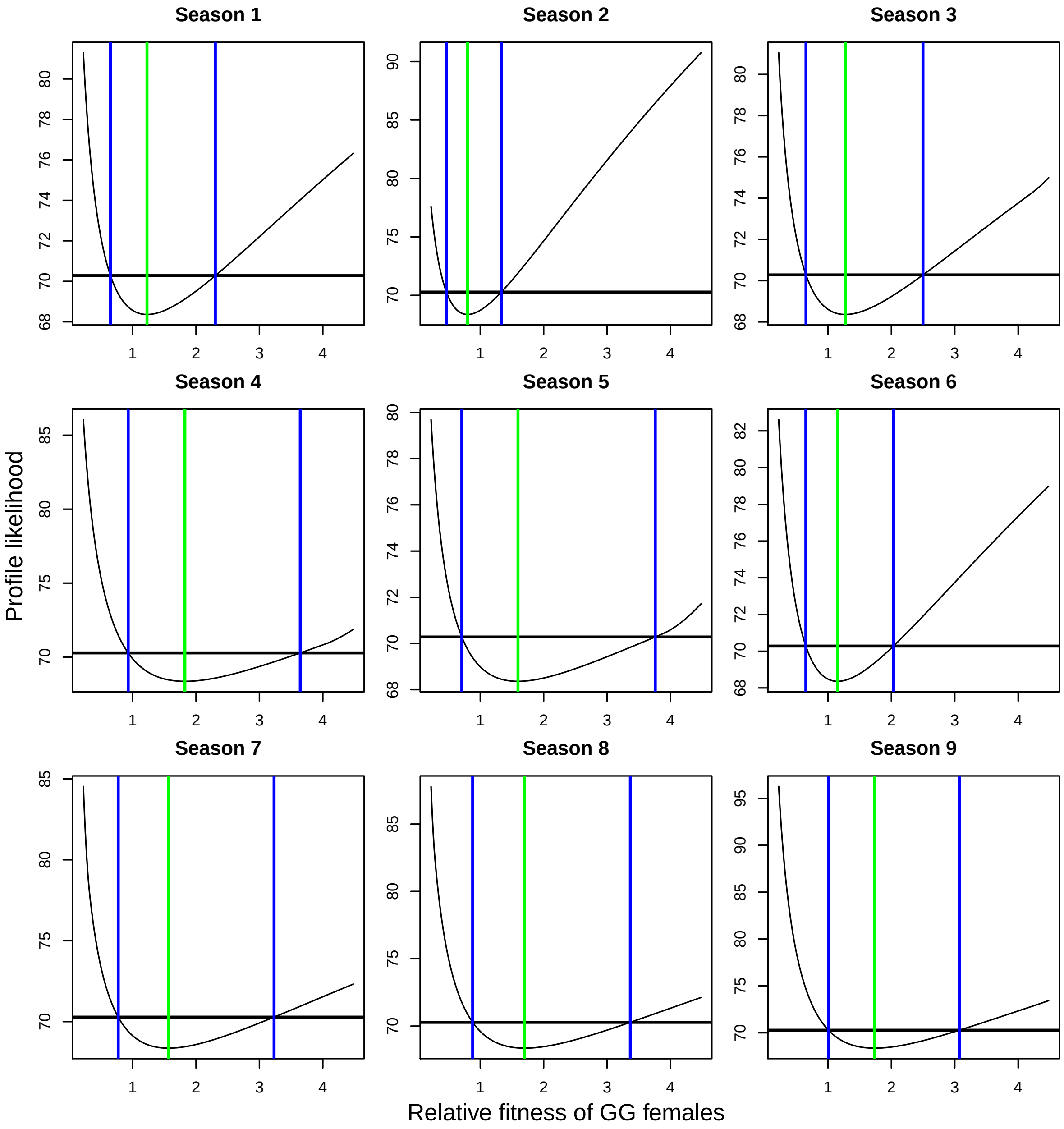

Figure S6 Likelihood profiles for the fitness of GG females in each season, $w_{GG,i}$. The value on the y-axis is the minimum negative log-likelihood with the focal parameter fixed at the value indicated on the x-axis. The green vertical lines indicate the least squares estimate, which is equal to the maximum likelihood estimate. The horizontal black line indicates the cutoff value for the confidence interval and the vertical blue lines indicate the upper and lower boundaries of the profile likelihood confidence intervals.

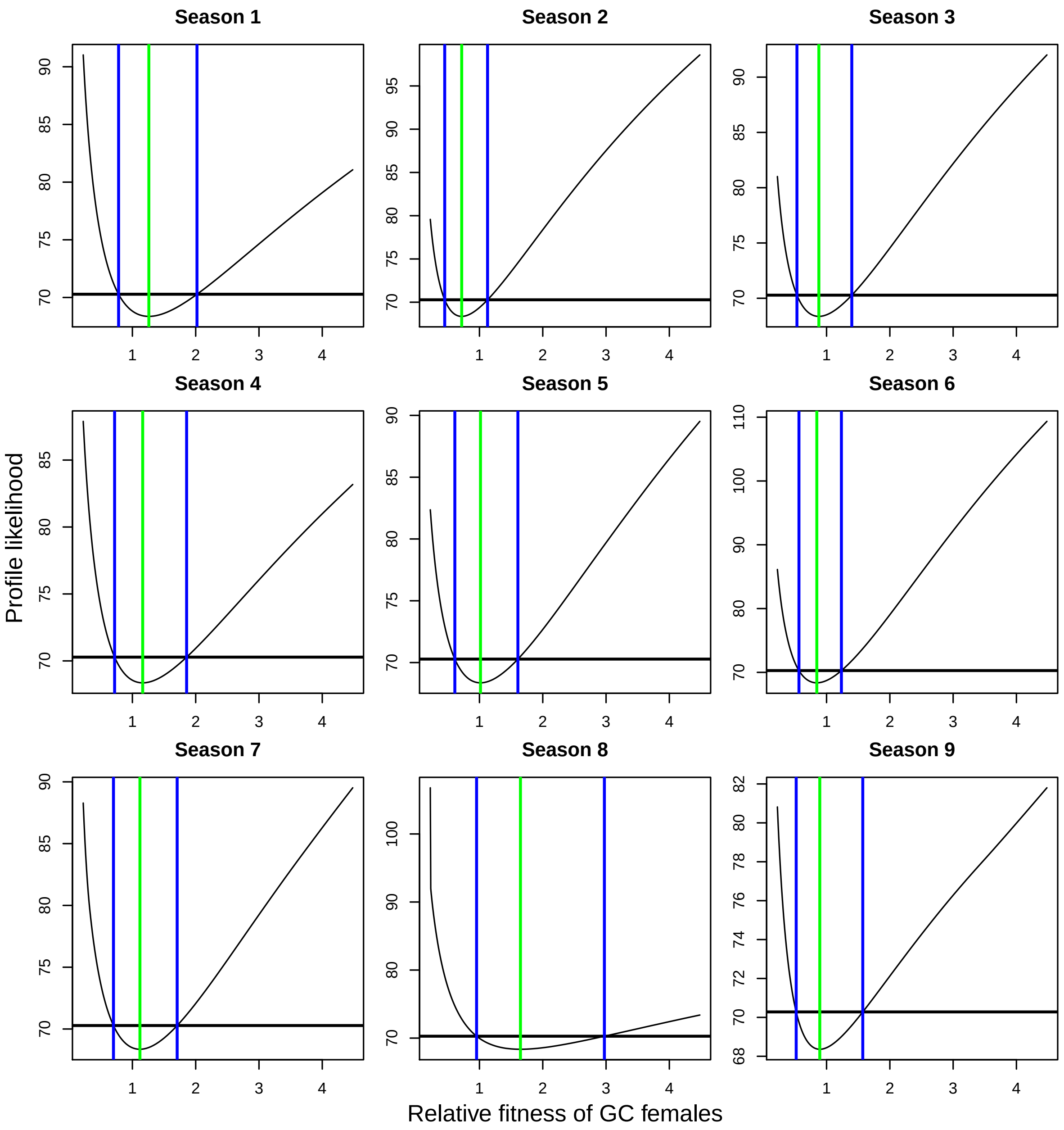

Figure S7 Likelihood profiles for the fitness of GC females in each season, $w_{GC,i}$. The value on the y-axis is the minimum negative log-likelihood with the focal parameter fixed at the value indicated on the x-axis. The green vertical lines indicate the least squares estimate, which is equal to the maximum likelihood estimate. The horizontal black line indicates the cutoff value for the 95% confidence interval and the vertical blue lines indicate the upper and lower boundaries of the corresponding profile likelihood confidence intervals.

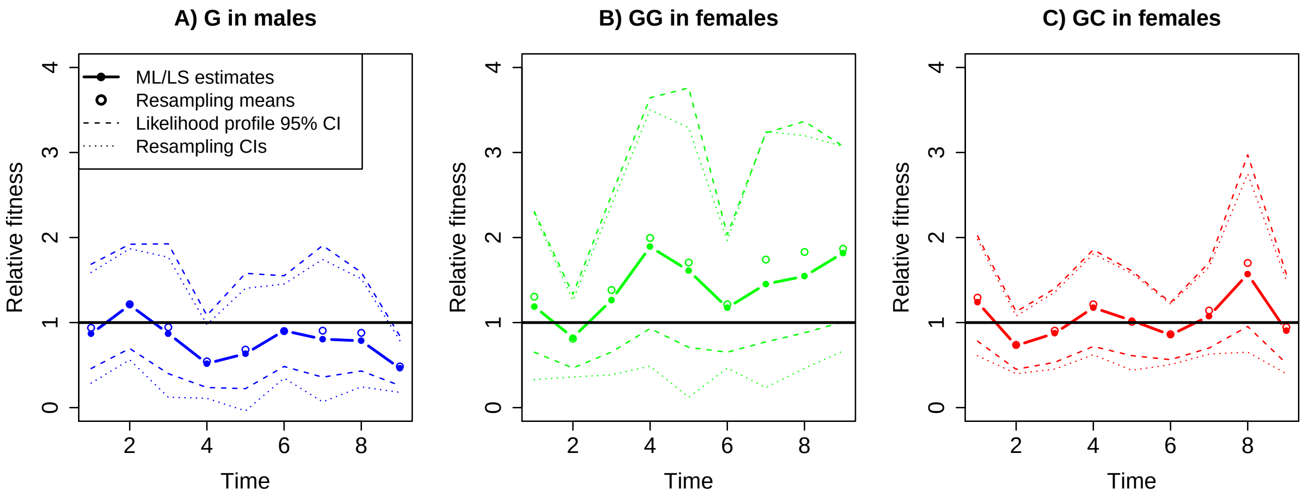

Figure S8 Comparison of 95% likelihood profile confidence intervals and mean ± 2 standard deviations of resampling parameter estimates.

Table S6 Model selection summary table

Model	Number of parameters	AIC
Full	30	196.7
Without temporally fluctuating selection	6	185.7
Only fluctuating selection (G dominant)	12	181.2
Only fluctuating selection (G recessive)	12	184.3
Fluctuating selection with fixed factors	14	183.7
Seasonal selection with fixed factors	7	187.0
Neutral	3	207.3

females + 2 constant fitness factors). The seasonal selection model with fixed factors was similar to the fluctuating selection model with fixed factors, but assumed that there are just two w_{GG} values, one for all odd-numbered seasons and one for all even-numbered seasons. This model had 7 parameters. Finally, the neutral model just had the 3 initial frequency parameters and assumed $w_{m,i} = w_{GG,i} = w_{GC,i} = 1$ for all seasons.

Based on the AIC values, the best model is the one with only fluctuating selection and G dominant in females. The estimates for the relative G fitness under this model are shown in Fig. S9 and the fit to the observed data is shown in Fig. S10.

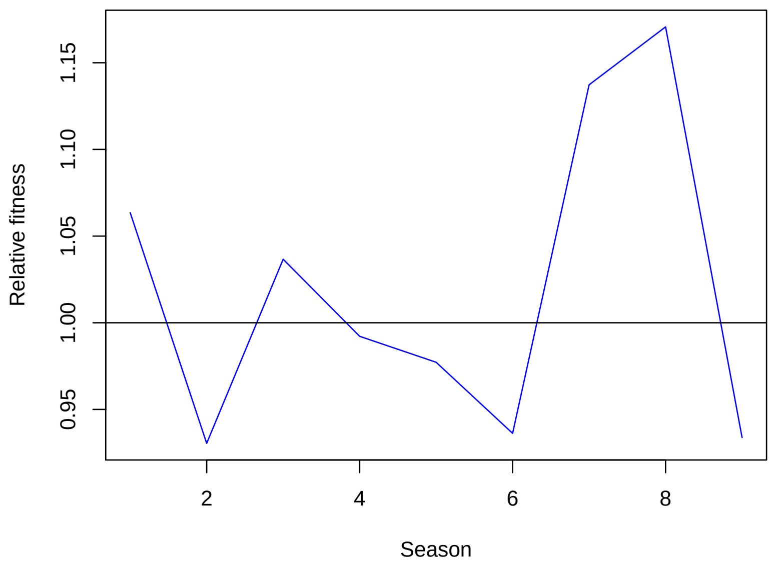

Figure S9 Relative fitness estimates under the model with only fluctuating selection and G dominant.

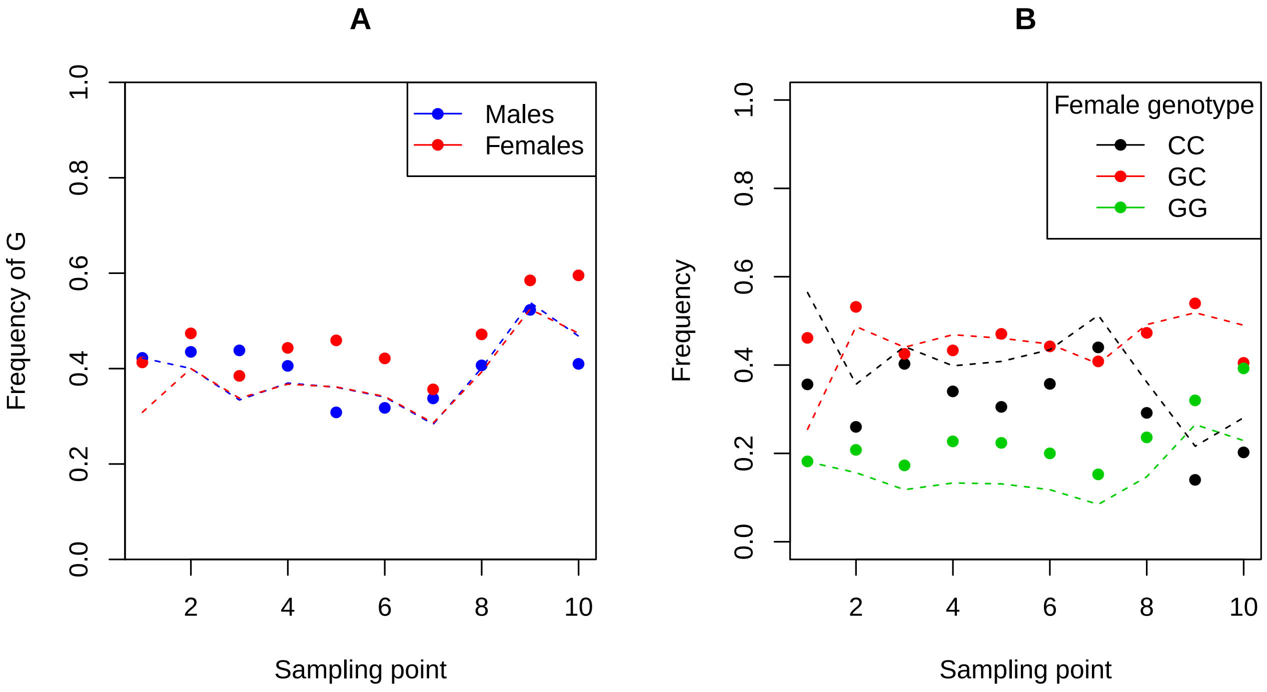

Figure S10 Fit of the model with only fluctuating selection and G dominant, the model with the lowest AIC, to the observed allele frequencies (A) and female genotype frequencies (B).

Long-term projections

We took the parameter estimates from Fig. S1 and iterated the dynamics for a large number of seasons to see whether polymorphism is maintained in the long run. This was done for a cycle repeating seasons 1 to 8 (Fig. S11 and for a season cycle repeating seasons 2 to 9 (Fig. S12). Note that in both cases, one season is left out to balance the number of summer and winter generations. Both figures are for five generations per season, but the results for the other season lengths are similar.

In all cases we tried, the fecundity selection model with its estimated parameters predicts long-term stable polymorphism (upper rows in Figs. S11 and S12), but the viability-selection model with its estimated parameters predicts that the C allele goes extinct in the long run. This difference appears to be attributable to the estimated parameter values. With the G allele dominant, the viability-selection model can also maintain polymorphism (see Fig. 2D in the main text).

To determine the role of sexually antagonistic vs. fluctuating selection in maintaining polymorphism, we also ran long-term predictions with all parameters constant over time and set to the average of their estimated values for the nine seasons (Table S7). Again, we obtain stable polymorphism in the fecundity-selection model, but not in the viability-selection model (Fig. S13).

Table S7 Average value of the estimated fitness parameters over time in the fecundity-selection and viability-selection model with 5 generations per season

Fitness parameter	Fecundity	Viability
w_m	1.80	0.79
w_{GG}	0.61	1.43
w_{GC}	1.12	1.06

Both sets of parameters in Table S7 display sexual antagonism with one allele favored in males and one allele favored in females. In both cases, the fitness of the GC heterozygotes is close to 1, i.e. close to that of the CC genotype. This means that with the average parameters in the fecundity-selection model, the allele that is advantageous in males is recessive in females. With the average parameters in the viability-selection model, the allele that is advantageous in males is dominant in females. If we change the average parameters estimated so that the male-beneficial allele is partially recessive, polymorphism becomes unstable in the fecundity-selection model where G is favored in males and stable in the viability-selection model where G is favored in females (Fig. S14). This is consistent with theoretical results by Patten and Haig (2009) who show that the more recessive the male-beneficial allele is, the easier it is to maintain a sexually antagonistic polymorphism on the X chromosome.

We also ran long-term simulations with the best model based on AIC. Also here, we did not observe maintenance of polymorphism (Fig. S15).

Finally, we studied how the uncertainty in the parameter estimates propagates into the predictions for long-term allele-frequency trajectories and maintenance of polymorphism. Again, we focused on the viability-selection model with 5 generations per season.

We generated 20,000 random parameter sets. To balance summer and winter generations, we again used seasons 1 to 8 in half of the sets and seasons 2 to 9 in the other half. For each parameter set, we drew its 24 fitness parameters (fitness of 3 genotypes in 8 seasons) from a vector of 100

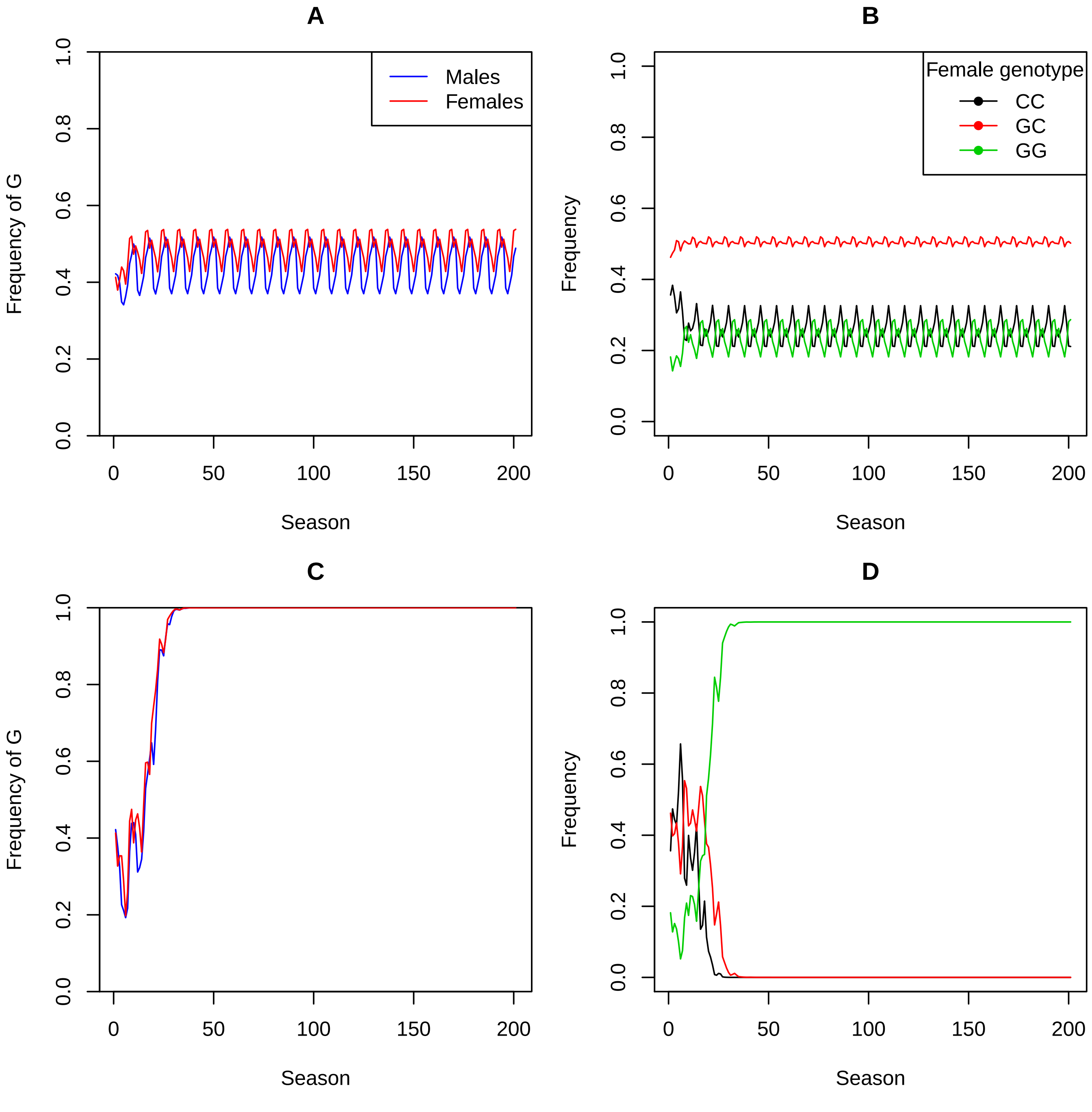

Figure S11 Long-term projections with the parameter estimates assuming 5 generations per season and using the season cycle 1,2,3,4,5,6,7,8. A, B: Results for the fecundity-selection model. C, D: Results for the viability-selection model.

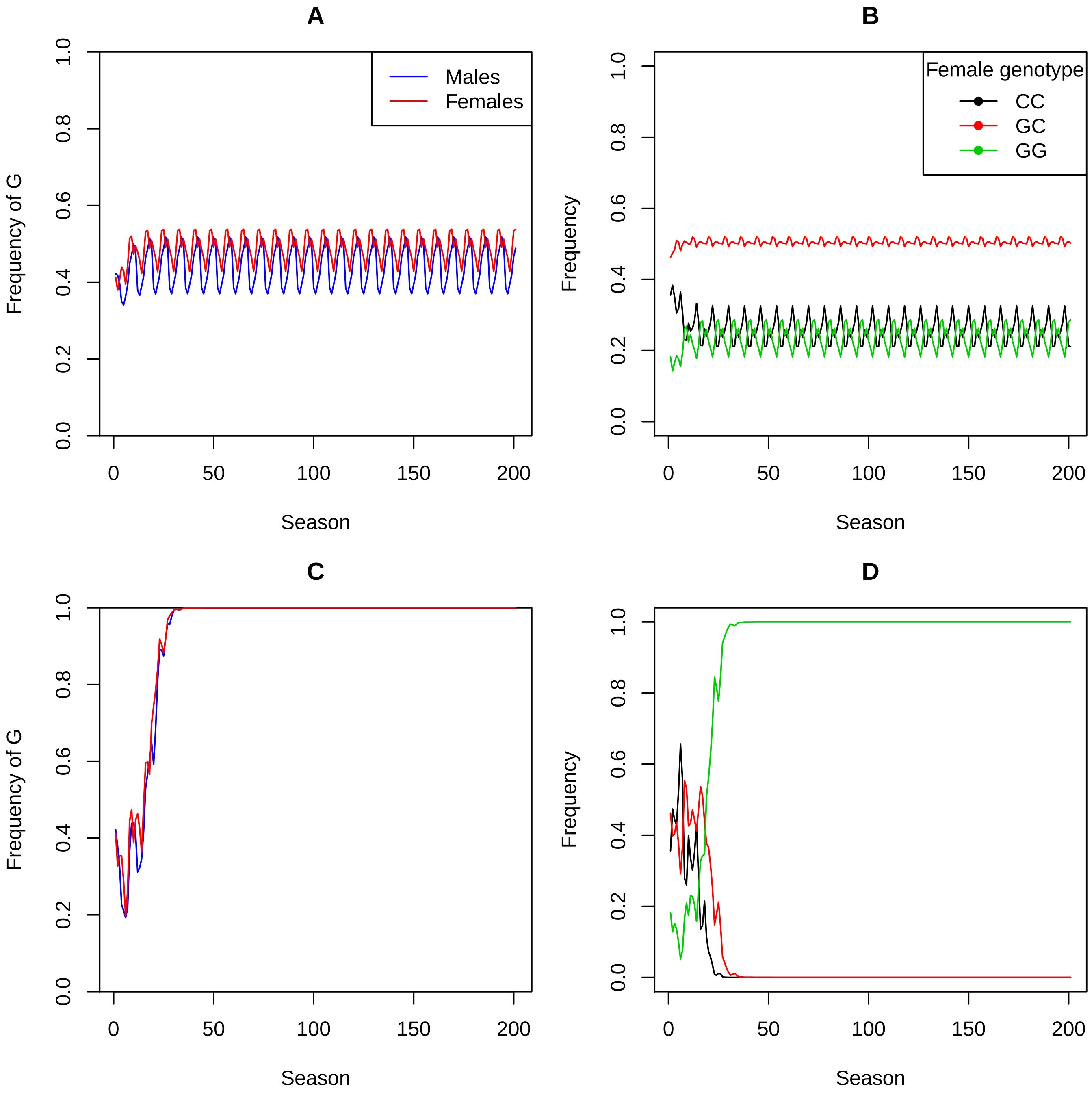

Figure S12 Long-term projections with the parameter estimates assuming 5 generations per season and using the season cycle 2,3,4,5,6,7,8,9. A, B: Results for the fecundity-selection model. C, D: Results for the viability-selection model.

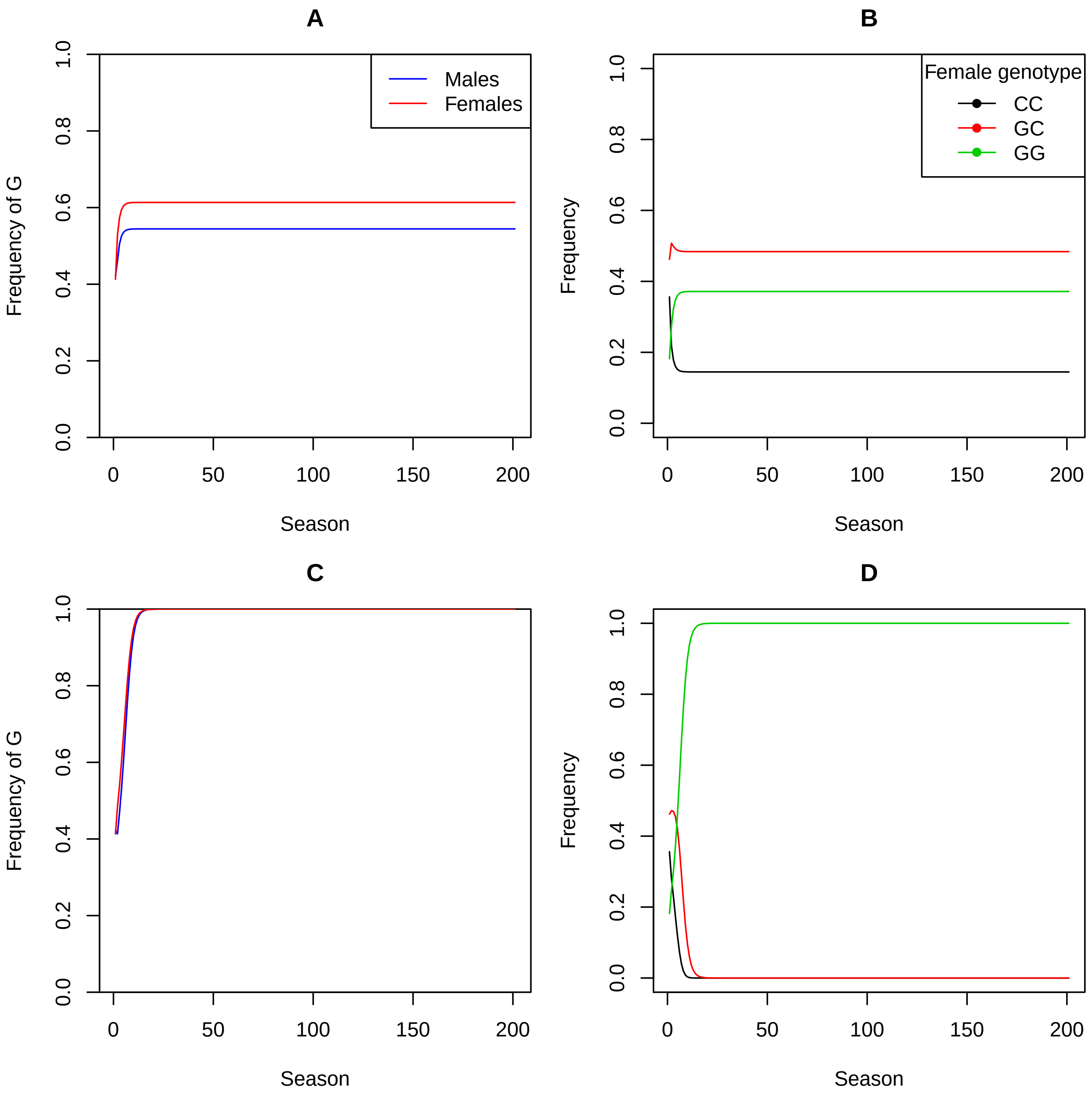

Figure S13 Long-term projections with the fitness values constant over time and given by the average parameter estimates in Table S7. A, B: Results for the fecundity-selection model. C, D: Results for the viability-selection model.

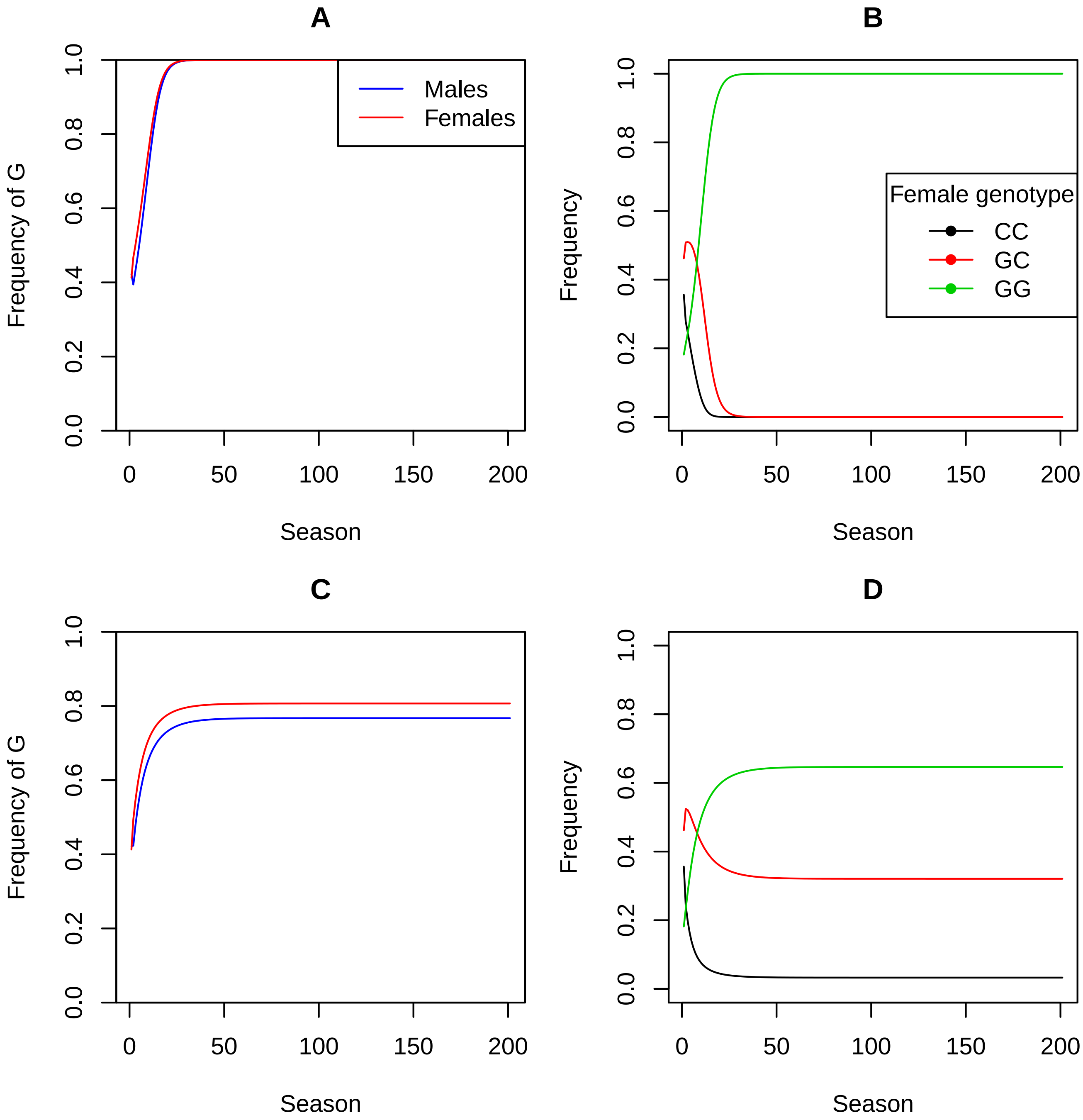

Figure S14 Long-term projections with the fitness values constant over time and given by the average parameter estimates in Table S7, but with dominance 0.715 ($w_{GC} = 1 + h * (w_{GG} - 1)$), such that the G allele is partially dominant in females. A, B: Results for the fecundity-selection model. C, D: Results for the viability-selection model.

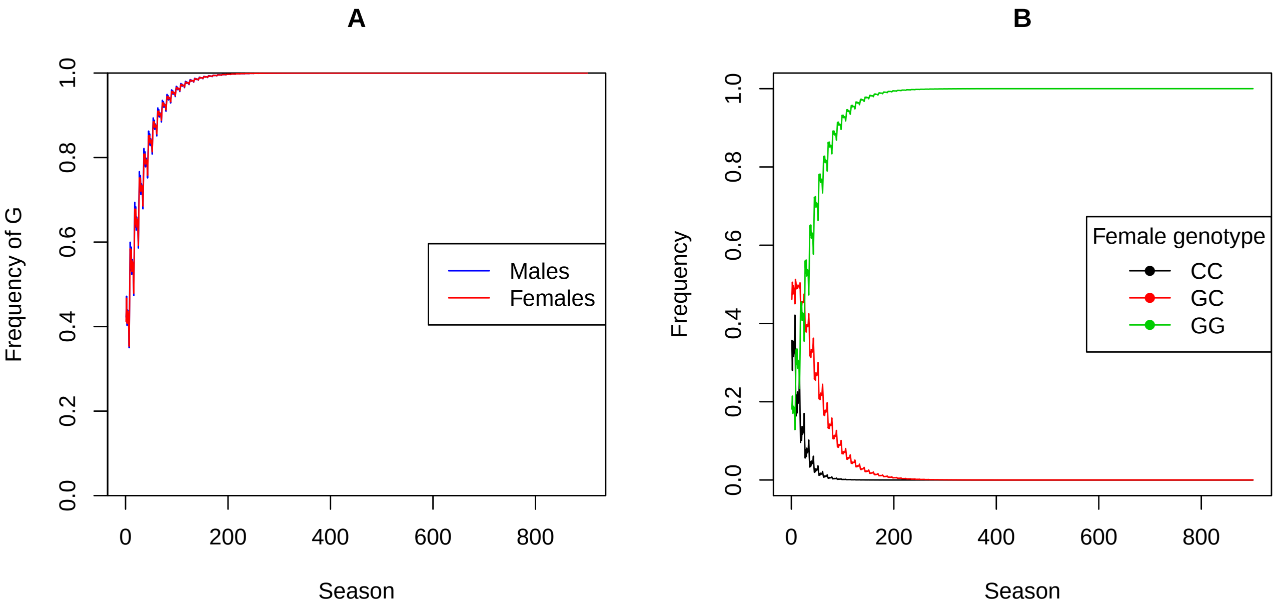

Figure S15 Long-term predictions for allele frequencies (A) and female genotype frequencies (B) under the model with only fluctuating selection and G dominant, the model with the lowest AIC.

values between exp(−1.5) and exp(1.5), evenly spaced on a logarithmic scale. The probability of a fitness value to be chosen was proportional to the profile likelihood for the respective genotype and season (see Figs. S5, S6, S7, but note that these figures show the negative log-likelihood).

For each parameter set, we then ran the model for 500 seasons, with every cycle of 8 seasons having the same parameter values. If the G allele frequency at the end of the 500 seasons was between 0.01 and 0.99, we considered polymorphism to be maintained. This was the case in 8.3% of parameter sets (Fig. S16).

For those parameter sets maintaining polymorphism, we additionally checked whether polymorphism depended on temporal fluctuations. For this, we kept the fitness values of the three genotypes constant over time and set them equal to their respective temporal average. In 42 % of cases that maintained polymorphism, polymorphism was still maintained with constant fitness values. The average fitness of GC females in these parameter sets (Fig. S16) was very similar to the average fitness of GG females, whereas in parameter sets that maintained polymorphism with fluctuating selection but not without, the average fitness of GC females was rather intermediate between CC and GG. Finally, in those parameter sets that did not maintain polymorphism, the average fitness of GC females was similar to CC females.

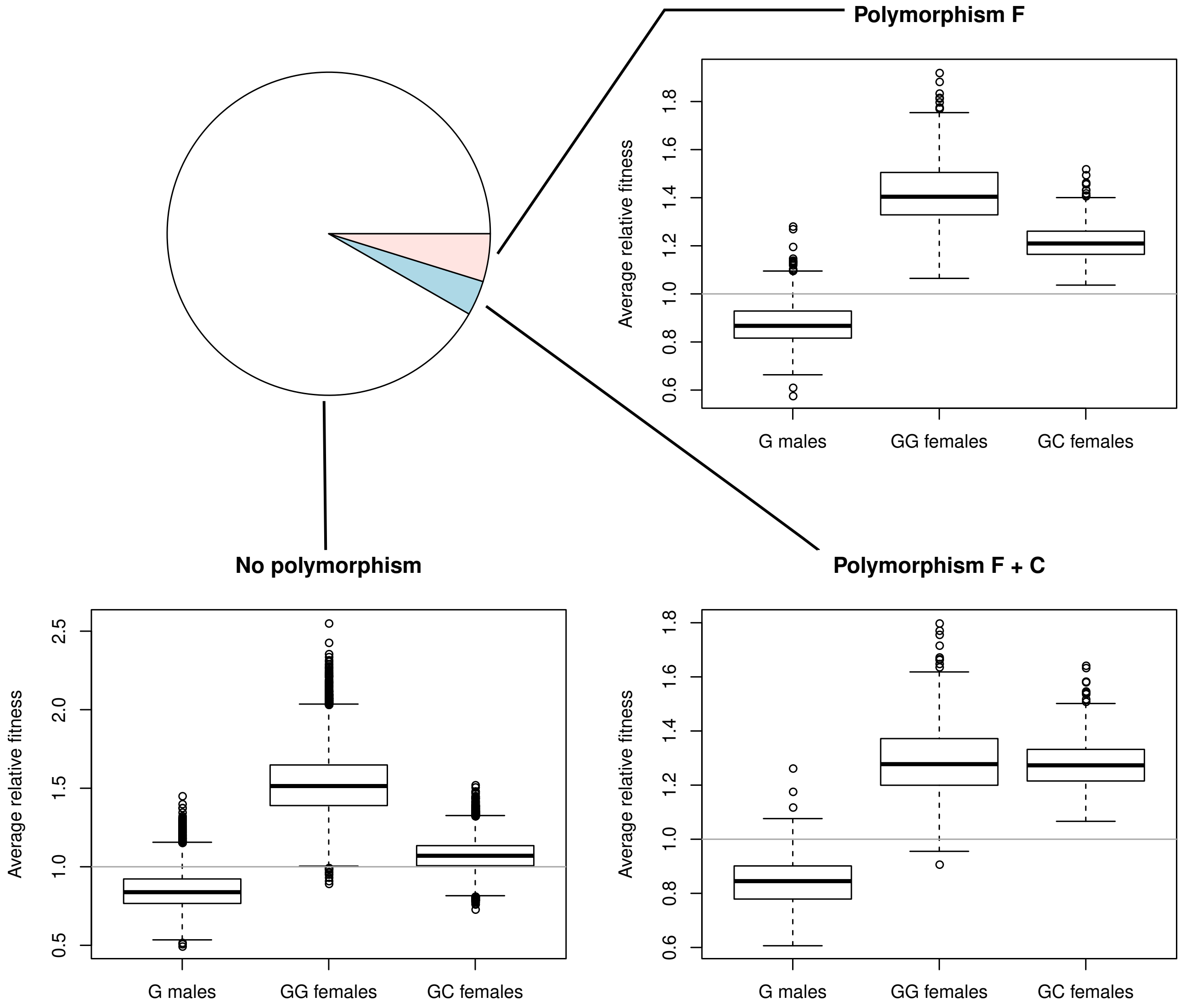

Figure S16 Long-term predictions taking into account uncertainty in the parameter estimates. The pie chart displays the proportion of parameter sets for which polymorphism was not maintained (white, 91.7%), maintained only in the full with temporal fluctuations (F, pink, 4.8%) or maintained in both the full model and the model with constant fitnesses set equal to the mean fitness of the respective genotype (F+C, blue, 3.5). The boxplots show the respective distributions of time-averaged fitness values.

References

Bolker, B., 2008 *Ecological Models and Data in R.* Princeton University Press, Princeton, Oxford.

Patten, M. M., and D. Haig, 2009 Maintenance or loss of genetic variation under sexual and parental antagonism at a sex-linked locus. Evolution 63: 2888–2895.
