# Supplementary material for: Sexual Antagonism, Temporally Fluctuating Selection, and Variable Dominance Affect a Regulatory Polymorphism in Drosophila melanogaster
Source: Mol Biol Evol. 2021 Jul 21;38(11):4891–907. doi: 10.1093/molbev/msab215 (PMC8557461; doi:10.1093/molbev/msab215)
Supplement: msab215_Supplementary_Data [file msab215_supplementary_data.zip › SuppText2.pdf]

Supplementary Text 2: Supplementary Methods

Sexual antagonism, temporally fluctuating selection, and variable dominance affect a regulatory polymorphism in *Drosophila melanogaster*

Amanda Glaser-Schmitt, Meike J. Wittmann, Timothy J. S. Ramnarine & John Parsch

DNA extraction and SNP genotyping of position 67

All larvae and flies were stored at -80 °C before DNA extraction. Flies or larvae for use in expression assays were stored in RNA/DNA shield (Zymo Research Europe; Freiburg, Germany), while flies or larvae for use in larval volume or body size and wing load assays were stored in 96% ethanol. For flies and larvae used in expression, larval volume, or body size and wing load assays, DNA was extracted from either the larval head (ending just below the mouth hooks) or adult leg using the Animal Direct PCR kit (Thermo Fisher Scientific; Waltham, Massachusetts, USA). For flies used in starvation resistance assays, DNA was extracted by homogenization in squishing buffer (10mM Tris-HCL pH = 8.0, 1mM EDTA, 25mM NaCl, 200 µg/ml Proteinase K) followed by a 30 minute incubation at 37 °C for protein digestion and 3 minutes at 95 °C to inactivate the Proteinase K. Genomic DNA from wild-caught flies was extracted using the MasterPure DNA Purification Kit (Lucigen; Middleton, Wisconsin, USA). For genotyping, a ~1.4-kb fragment surrounding the SNP at position 67 was amplified using the following primers (5'–3'): TCGTGATGATGCCGATTCAGA and CCCGATCCTCCCAGCATTTT, followed by digestion with *Hha*I (New England Biolabs; Ipswich, Massachusetts, USA), which cuts the C but not the G variant into two fragments of ~925 and ~450 bp. It should be noted that *fiz* is encoded on the minus strand and throughout the text we refer to the SNP variants in *fiz*'s coding direction. Therefore, according to the coordinates of the reference genome (release 6; Gramates et al. 2017), the derived variant at position 67 (coordinate X:14,909,071) is a C and the ancestral variant is a G.

Larval and adult staging

Because temperature and density during rearing as well as age can affect body size and wing loading (Santos et al. 1994; James and Partridge 1995; Angilletta et al. 2004) as well as expression and starvation resistance, all adult flies and larvae used in phenotypic or expression assays were strictly controlled. All staging was performed at 25 °C with a 14 hr

light:10 hr dark cycle. Adults were placed in cages and allowed to lay eggs on molasses-agar plates supplemented with yeast for ~60 hours. For adult staging, first instar larvae were collected and transferred to small vials containing cornmeal-molasses medium at a density of 50 larvae per vial. After eclosion, flies were allowed to mate and aged to one-day-old for expression, body size, and wing loading assays or 5-days-old for starvation resistance assays. For larval staging, first instar larvae were transferred to large vials containing cornmeal-molasses medium supplemented with 5% bromophenol blue (Carl Roth; Karlsruhe, Germany) at a density of 250 larvae per vial. Late third instar wandering larvae (shortly before pupariation, puff stages 7–9) were identified by the clearing of their guts. Female larvae were identified by the size of their gonads and were washed in PBS and gently dried and then kept on ice until use. Larvae for RNA extraction were stored at -80 °C before use.

Expression analysis

We surveyed *fiz* expression in whole female F2 flies and larvae, with a leg or the head removed, respectively, for genotyping. Total RNA was extracted from individual one-day-old adult females or late third instar wandering larvae, and a DNAse I digestion was performed using the MasterPure RNA Purification Kit (Lucigen; Middleton, Wisconsin, USA). Using random hexamer primers and Superscript III reverse transcriptase (Invitrogen; Carlsbad, California, US), 2–3μg total RNA for each sample was reverse transcribed following the manufacturer's protocol. TaqMan Gene Expression Assays (Invitrogen; Carlsbad, California, USA) were then performed on the resulting cDNA using probes specific to *fiz* (Dm01838873_g1) and the ribosomal protein gene *RpL32* (Dm02151827_g1), which was used as an endogenous control. We surveyed *fiz* expression in 4–6 larvae or flies for each genotype in each cross pair. The ΔΔCt method was used to calculate normalized gene expression (Pfaffl 2001). Briefly, for each sample, the average threshold cycle (Ct) of 2 technical replicates was measured, and ΔCt was calculated as the mean Ct difference between the probe of interest and the *RpL32* probe. The fold-change difference in expression relative to the CC genotype from the reciprocal NL crosses (see Crosses to test the association of variation at position 67 with phenotype section) was calculated as $2^{-(\Delta CtX - \Delta CtY)}$, where ΔCtX is the mean ΔCt value for each sample of interest and ΔCtY is the mean ΔCt value of the CC genotype from the reciprocal NL crosses.

Reporter gene assays

We surveyed reporter gene expression in whole female flies and late wandering third instar larvae. For each reporter gene strain or F1 hybrid, β-galactosidase activity was measured in groups of 15 adult females or 8 female late wandering third instar larvae for 4–6 biological replicates per developmental stage. Soluble proteins were extracted, and a β-galactosidase activity assay was performed as described in Glaser-Schmitt and Parsch (2018). β-galactosidase activity was measured spectrophotometrically by following the change in absorbance at 420 nm at 37 °C.

Body size and wing load assays

We surveyed body size and wing load in individual female F2 flies from the MU crosses. For each genotype, we measured wet weight, wing length and area, and wing load index for 7–27 flies. Individual flies were anesthetized with CO_2 and the wet weight was measured by placing the fly in a pre-weighed 1.5 mL Eppendorf tubes on ice for 5 minutes before being weighed on a Mettler H51 scale (d = 0.01 mg, error = 0.05 mg). The weight of a fly was then calculated as the weight of the fly and tube minus the weight of the tube. For each fly, the right wing (or the left wing if the right wing was damaged) was then dissected in isopropanol, mounted in Euparal (Carl Roth; Karlsruhe, Germany), and allowed to dry at least 1 week before being photographed. Wings were photographed using a Nikon D3300 camera and compound microscope. Images were analyzed in ImageJ (Schneider et al. 2012). A piece of millimeter paper was included in all images for scale. Wing length was measured in a straight line from the humeral-costal break to the third longitudinal vein, and wing area was estimated as previously described (Gilchrist and Partidge 1999). Wing load index was calculated as the wet weight of a fly divided by the area of its wing.

Larval volume assays

Larval volume was measured in F2 late wandering third instar larvae (116 hours after egg laying) for 10–17 larvae per genotype and cross. Larvae were placed on ice for at least 5 minutes then photographed using a Nikon D3300 camera and a compound microscope, and images were analyzed in ImageJ (Schneider et al. 2012). A piece of millimeter paper was included in all images for scale. Larval volume was calculated as $4/3\pi(L/2)^2(d/2)$, where L = length and d = diameter (Colombani et al. 2005).

References

Angilletta MJ, Steury TD, Sears MW. 2004. Temperature, growth rate, and body size in ectotherms: Fitting pieces of a life-history puzzle. Integr Comp Biol. 44(6):498-509.

Colombani J, Bianchini L, Layalle S, Pondeville E, Dauphin-Villemant C, Antoniewski C, Carré C, Noselli S, Léopold P. 2005. Antagonistic actions of ecdysone and insulins determine final size in *Drosophila*. Science 310(5748):667-670.

Gilchrist AS, Partridge L. 1999. A comparison of the genetic basis of wing size divergence in three parallel body size clines of *Drosophila melanogaster*. Genetics 153(4):1775-1787.

Glaser-Schmitt A, Parsch J. 2018. Functional characterization of adaptive variation within a *cis*-regulatory element influencing *Drosophila melanogaster* growth. PLoS Biol. 16(1):e2004538.

Gramates LS, Marygold SJ, Santos GD, Urbano JM, Antonazzo G, Matthews BB, Rey AJ, Tabone CJ, Crosby MA, Emmert DB et al. 2017. FlyBase at 25: Looking to the future. Nucleic Acids Res. 45(D1):D663-D671.

James AC, Partridge L. 1995. Thermal evolution of rate of larval development in *Drosophila melanogaster* in laboratory and field populations. J Evol Biol. 8(3):315-330.

Pfaffl MW. 2001. A new mathematical model for relative quantification in real-time RT–PCR. Nucleic Acids Res. 29(9):e45.

Santos M, Fowler K, Partridge L. 1994. Gene-environment interaction for body size and larval density in *Drosophila melanogaster*: An investigation of effects on development time, thorax length and adult sex ratio. Heredity 72(5):515-521.

Schneider CA, Rasband WS, Eliceiri KW. 2012. NIH image to ImageJ: 25 years of image analysis. Nat Methods. 9(7):671-675.
